# Repositioning tolcapone as a potent inhibitor of transthyretin amyloidogenesis and associated cellular toxicity

**DOI:** 10.1038/ncomms10787

**Published:** 2016-02-23

**Authors:** Ricardo Sant'Anna, Pablo Gallego, Lei Z. Robinson, Alda Pereira-Henriques, Nelson Ferreira, Francisca Pinheiro, Sebastian Esperante, Irantzu Pallares, Oscar Huertas, Maria Rosário Almeida, Natàlia Reixach, Raul Insa, Adrian Velazquez-Campoy, David Reverter, Núria Reig, Salvador Ventura

**Affiliations:** 1 Institut de Biotecnologia i Biomedicina and Departament de Bioquímica i Biologia Molecular, Universitat Autònoma de Barcelona, Bellaterra, Barcelona 08193, Spain; 2 Molecular and Experimental Medicine Department, The Scripps Research Institute, La Jolla, California 92037, USA; 3 Molecular Neurobiology, IBMC- Instituto de Biologia Molecular e Celular i3S - Instituto de Investigação e Inovação em Saúde and ICBAS- Instituto de Ciências Biomédicas de Abel Salazar, Universidade do Porto, Porto, Portugal; 4 SOM-Biotech, Baldiri Reixac 4, Barcelona 08028, Spain; 5 Institute of Biocomputation and Physics of Complex Systems (BIFI), Joint Unit IQFR-CSIC-BIFI, Universidad de Zaragoza, Zaragoza 50018, Spain; 6 Department of Biochemistry and Molecular and Cell Biology, University of Zaragoza, Zaragoza 50009, Spain; 7 Instituto de Investigación Sanitaria de Aragón (IIS Aragón), Zaragoza 50009, Spain; 8 Fundación ARAID, Government of Aragón, Zaragoza 50003, Spain

## Abstract

Transthyretin (TTR) is a plasma homotetrameric protein implicated in fatal systemic amyloidoses. TTR tetramer dissociation precedes pathological TTR aggregation. Native state stabilizers are promising drugs to treat TTR amyloidoses. Here we repurpose tolcapone, an FDA-approved molecule for Parkinson's disease, as a potent TTR aggregation inhibitor. Tolcapone binds specifically to TTR in human plasma, stabilizes the native tetramer *in vivo* in mice and humans and inhibits TTR cytotoxicity. Crystal structures of tolcapone bound to wild-type TTR and to the V122I cardiomyopathy-associated variant show that it docks better into the TTR T_4_ pocket than tafamidis, so far the only drug on the market to treat TTR amyloidoses. These data indicate that tolcapone, already in clinical trials for familial amyloid polyneuropathy, is a strong candidate for therapeutic intervention in these diseases, including those affecting the central nervous system, for which no small-molecule therapy exists.

## Abstract (web summary)

Misfolding of transthyretin can cause amyloid aggregation disorders that can be treated by stabilizing the tetrameric form with tafamidis. Here the authors show that tolcapone, a drug already FDA-approved for Parkinson disease, has strong transthyretin stabilizing function and might be a superior therapeutic option for CNS amyloidosis as it can cross the blood brain barrier.

## Graphical abstract

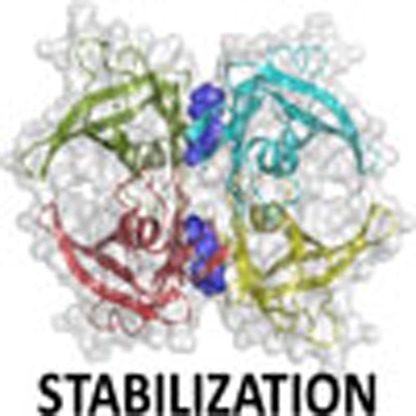

The misassembly of soluble proteins into toxic aggregates, including amyloid fibrils, underlies a variety of human diseases^1^. Transthyretin (TTR) is a homotetrameric protein produced mainly in the liver and in the brain's choroid plexus, and circulates in plasma and cerebrospinal fluid^2^,^3^. TTR aggregation is associated with senile systemic amyloidosis (SSA)^3^, familial amyloid cardiomyopathy (FAC)^4^ and familial amyloid polyneuropathy (FAP)^5^. SSA and FAC are caused by aggregation and deposition of wild-type and mutant TTR, respectively, preferentialy in the heart. FAP is characterized by mutant TTR deposition in peripheral and autonomic nerves and the heart, but also in other sites such as the lung, carpal tunnel and gut. More than 100 different TTR variants have been reported^6^. For some rare TTR mutations aggregation develops in the central nervous system, resulting in amyloid deposits in the leptomeninges, in the brain parenchyma, and in the eyes^7^,^8^,^9^.

Plasma TTR binds and transports holo retinol binding protein and thyroxine (T_4_), whereas in the cerebrospinal fluid it transports T_4_ only^10^. TTR is composed of four identical 127 amino-acid residue β-sheet-rich subunits, termed A, B, C and D^11^. The TTR tetramer is formed by association of the AB and CD dimers. The weaker dimer–dimer interface defines two, largely unoccupied (<1% T_4_ bound), funnel-shaped T_4_-binding sites^12^. Tetramer dissociation is the rate-limiting step for TTR aggregation^13^,^14^. Accordingly, autosomal dominant mutations often destabilize TTR tetramer, thus increasing amyloidogenesis^15^,^16^.

For many years, liver or combined liver and heart transplantation were the only palliative treatments for the TTR amyloidoses^17^. More recently, it has been shown that small molecules able to bind to the TTR T_4_-binding sites increase the energy barrier of tetramer dissociation, acting as kinetic stabilizers, thus stalling TTR aggregation^18^,^19^,^20^,^21^,^22^,^23^,^24^,^25^,^26^,^27^,^28^. A pharmacologic strategy, based on stabilization of the TTR native tetramer by the benzoxazole tafamidis has been approved in Europe and Japan for the treatment of early-stage FAP^29^,^30^. However, tafamidis might not be potent enough to treat advanced TTR amyloidoses^31^. The lack of an Food and Drug Administration (FDA)-approved candidate for the treatment of TTR amyloidoses reflects the difficulty of moving from the discovery of *in vitro* hits to the development of clinically effective and safe drugs. Drug repositioning offers a potentially valuable and productive approach to identify candidates for new pharmacologic applications. The process involves the identification of existing compounds licensed for a different therapeutic indication^32^. Those candidates have already established safety profiles, reducing the time and cost required to bring them to trial and into the clinic for their new indication. Diflunisal, an FDA-approved nonsteroidal anti-inflammatory agent, acts as a TTR kinetic stabilizer^33^,^34^. Although its *in vitro* affinity for TTR T_4_-binding sites is significantly lower than that of tafamidis, a randomized clinical trial has shown that the diflunisal treatment of patients with FAP for 2 years reduced the rate of progression of neurological impairment^35^, demonstrating the validity of drug repurposing for TTR amyloidoses.

Here we identify tolcapone as a potential inhibitor of TTR amyloidogenesis. Tolcapone is an orally active cathecol-*O*-methyltransferase (COMT) inhibitor authorized in the United States and Europe as an adjunct to levodopa and carbidopa for the treatment of Parkinson's disease. We show that tolcapone binds specifically to TTR, stabilizes both wild-type (WT-TTR) and mutant TTR variants, reduces their aggregation and prevents TTR-induced cardiotoxicity more effectively than tafamidis. X-ray crystallography and calorimetric assays reveal the molecular determinants that account for the activity of tolcapone in both WT-TTR and the V122I cardiomyopathy-associated variant. The anti-aggregational properties of this repositioned drug make it an ideal candidate for the therapeutic treatment of the TTR amyloidoses.

## Results

### Tolcapone inhibits TTR aggregation

**Figure 1:**
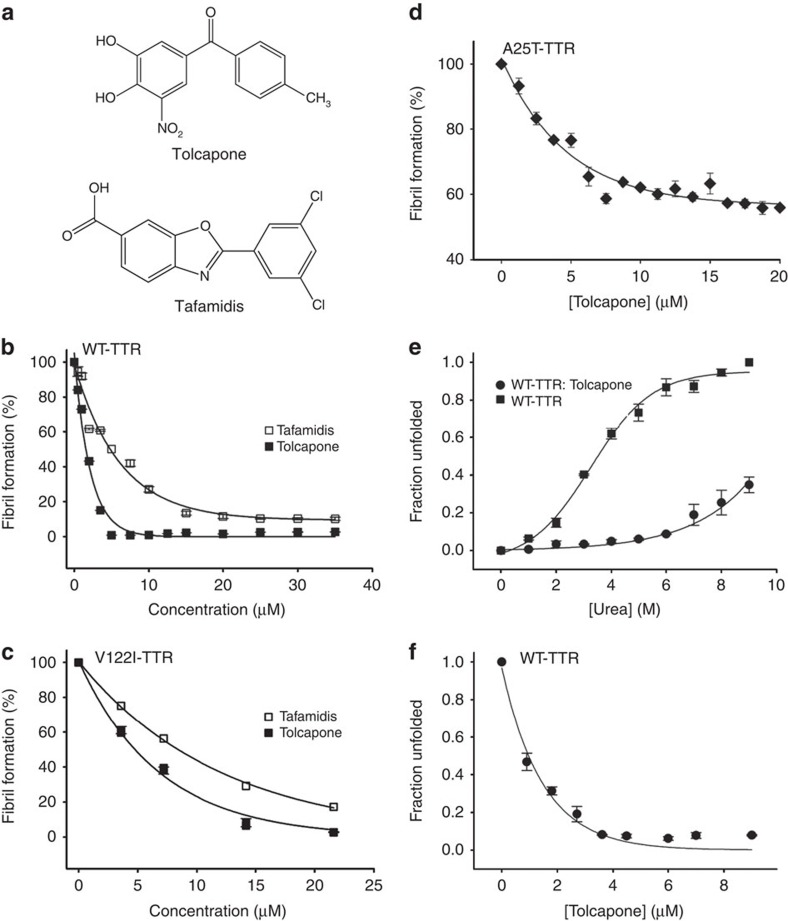
Tolcapone and tafamidis effects over WT, V122I-TTR and A25T-TTR aggregation and stability. (**a**) Chemical structures of the commercially available drugs tolcapone (Tasmar, CAS 134308-13-17) and tafamidis. Anti-amyloidogenic effect of tolcapone and tafamidis on (**b**) WT-TTR and (**c**) V122I-TTR and (**d**) anti-amyloidogenic effect of tolcapone on A25T-TTR. TTR solutions were incubated with several concentrations of test molecules and aggregation was induced by acidification. Turbidity at 340 nm was used to monitor TTR aggregation and fibril formation. At the end of the assays, the percentage of TTR aggregation for each experimental condition was calculated with respect to the TTR turbidity values obtained in the absence of compounds (vehicle only). (**e**) WT-TTR was incubated in the presence or absence of 10 molar equivalents of tolcapone. Urea was added to promote protein unfolding at the indicated final concentrations. (**f**) WT-TTR was incubated with several concentrations of tolcapone (from 0.25 to 10 molar equivalents with respect to WT-TTR) and denaturation promoted by addition of urea at a final concentration of 6.5 M. In both assays **e** and **f**, after 72 h incubation at RT, Trp fluorescence intensity (355/335 nm) was measured as a sensor of tetramer integrity. TTR stabilization effect induced by tolcapone binding was calculated with respect to the tryptophan fluorescence emission of the protein in the absence of the compound (vehicle only). In all panels, error bars indicate the standard error of the mean (s.e.m.; *n*=3).

The Symyx Comprehensive Medicinal Chemistry database includes compounds under clinical development, phase II-III and molecules already approved for human use. A total of 30 compounds were selected for further experimental characterization (Supplementary Table 1). We first tested the anti-TTR aggregational activity of these molecules in the 0–40 μM range with the highly amyloidogenic Y78F-TTR variant at pH 4.2 (ref 36). This acidic pH induces TTR aggregation increasing thus sample turbidity and light scattering^15^. Supplementary Table 1 shows the obtained EC_50_ values for TTR aggregation inhibition. The most active compound exhibits an EC_50_ of 3.92±0.30 μM and corresponds to the commercial drug tolcapone (Tasmar, CAS 134308-13-17; Fig. 1a). Under the same conditions, tafamidis displays an EC_50_ of 5.36±0.57 μM. We validated these results by testing the capacity of tolcapone to prevent aggregation of WT-TTR and the FAC-associated V122I-TTR variant^37^ at pH 4.4 (refs 38, 39). Tolcapone was highly effective at inhibiting both WT-TTR and V122I-TTR aggregation with EC_50_ values of 1.50±0.12 and 4.72±0.45 μM, respectively, as measured by light scattering (Fig. 1b,c). Under the same conditions, tafamidis displayed EC_50_ values of 4.78±0.47 and 8.35±0.80 μM for WT-TTR and V122I-TTR, respectively (Fig. 1b,c). At any TTR/drug ratio tested, tolcapone exhibited a stronger aggregation inhibitory activity than tafamidis. We confirmed tolcapone's anti-aggregational activity using sedimentation assays (Supplementary Fig. 1a). The A25T-TTR variant is among the most destabilized and fastest dissociating TTR tetramers^8^; however, it only causes an amyloid pathology in the brain. Because of its instability, the protein is mostly degraded in the liver and not secreted into the bloodstream. The high concentration of T_4_ in the choroid plexus acts as a kinetic stabilizer allowing A25T-TTR folding and secretion^16^, but once secreted the tetramers dissociate, resulting in amyloid deposition in the leptomeninges. As tolcapone is able to cross the blood–brain barrier^40^, we tested whether it could reduce the aggregation of this variant *in vitro*. A25T-TTR (3.6 μM) was incubated in the presence or absence of tolcapone (0–20 μM) at pH 5.0 and 37 °C for 22 h, where this variant exhibits maximum amyloidogenicity^8^. A25T-TTR requires only 1 h to become fully aggregated due to its high kinetic unstability^8^. Four molar equivalents of tolcapone (15 μM) sufficed to reduce between 40% (Fig. 1d) and 50% (Supplementary Fig. 1b) of A25T-TTR aggregation, resembling the anti-aggregational activity exerted by T_4_ at the same pH (ref. 8).

### Tolcapone kinetically stabilizes TTR

We addressed whether the anti-aggregational effect of tolcapone was mediated by tetramer stabilization. In urea, TTR tetramer dissociation precedes monomer unfolding, a process easily tracked by monitoring changes in intrinsic Trp fluorescence^41^. WT-TTR was incubated in the presence or absence of tolcapone (10 molar equivalents with respect to TTR; that is, 5 molar equivalents of tolcapone per TTR-binding site) and increasing concentrations of urea (0–9 M) for 72 h. Tolcapone does not interfere with TTR Trp fluorescence (Supplementary Fig. 2), therefore, Trp fluorescence was measured to calculate the percentage of folded protein at any given urea concentration^15^. Tolcapone exerted a strong stabilizing effect, allowing more than 80% of TTR to remain in the native state upon incubation in 8 M urea (Fig. 1e).

At high denaturant concentrations, the rate of TTR tetramer dissociation is linked irreversibly to monomer unfolding. WT-TTR (1.8 μM) was incubated in the absence and presence of tolcapone (0.25–10 molar equivalents with respect to TTR) in 6.5 M urea at room temperature (RT). Intrinsic fluorescence was recorded after 72 h. In the presence of 2 and 4 molar equivalents of tolcapone, only, 33.8 and 8% of WT-tetramer, respectively, were unfolded, indicating a dose-dependent kinetic stabilization of TTR tetramer (Fig. 1f).

### Tolcapone binds with high affinity to TTR

The strong stabilizing and anti-aggregational activity of tolcapone suggest that it binds tightly to the TTR T_4_-binding sites. The ability of tolcapone and tafamidis to compete with T_4_ for TTR binding was assessed by a gel filtration assay^42^. Competition binding experiments using radiolabelled T_4_ and WT-TTR were performed in the presence of different concentrations of test compounds and the T_4_ fraction bound to TTR was separated from the unbound fraction by gel filtration. The EC_50_ values for tolcapone and tafamidis were 41.4 and 183.5 nM, respectively; thus tolcapone displaces radiolabelled T_4_ from TTR 4 times more efficiently than tafamidis.

**Figure 2:**
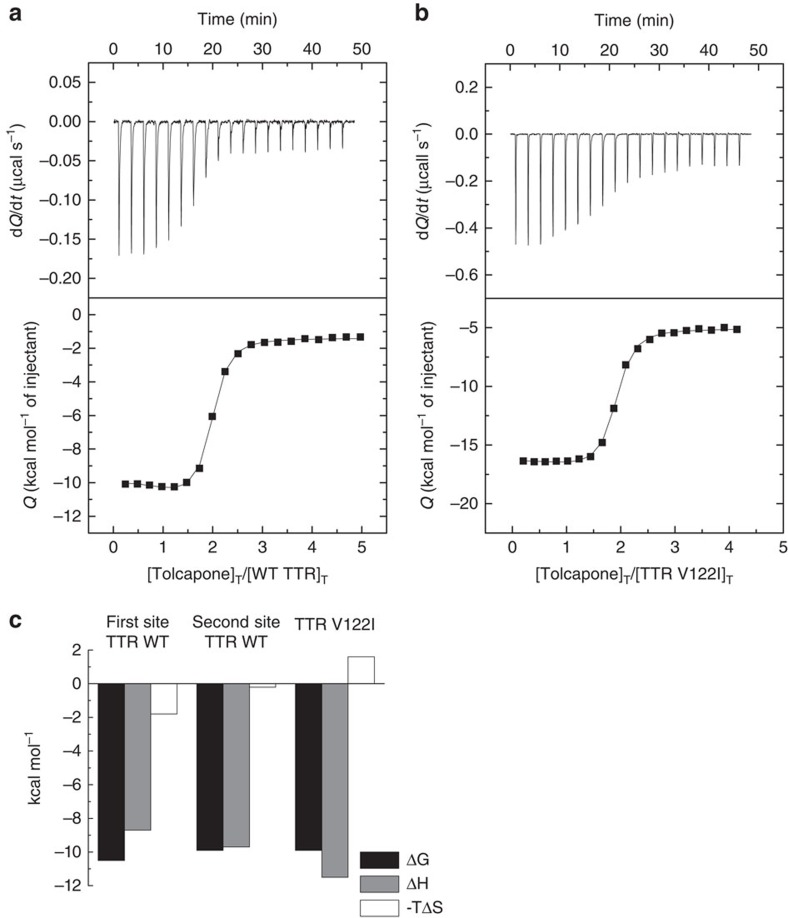
Interaction of TTR with tolcapone assessed by ITC. Top panels: Thermogram (thermal power versus time) after baseline correction; bottom panel: binding isotherm (normalized heat versus molar ratio of reactants). (**a**) WT and (**b**) V122I-TTR. The solid line corresponds to the best fit for each of the proteins. For WT-TTR, the data were fit by a general binding model for a protein with two ligand-binding sites. The non-linear regression analysis provided the estimations for the overall binding parameters: *β*_1_=9.3 × 10^7^ M^−1^, Δ*H*_1_=−8.7 kcal mol^−1^, *β*_2_=7.9 × 10^14^ M^−2^, Δ*H*_2_=−18.4 kcal mol^−1^. Nonlinear regression analysis indicated that V122I-TTR has two identical and independent binding sites for tolcapone and provided the estimations for the common binding parameters: *β*_1_=1.8 × 10^7^ M^−1^, Δ*H*_1_=−11.5 kcal mol^−1^. (**c**) Site-specific binding parameters for the first and second ligand bound to WT-TTR can be calculated from the overall binding parameters: Kd_1_=21 nM, Δ*h*_1_=−8.7 kcal mol^−1^, Kd_2_=58 nM, and Δ*h*_2_=−9.7 kcal mol^−1^. Common binding parameters for V122I-TTR sites were calculated to be Kd_1_=56 nM, Δ*h*_1_=−11.5 kcal mol^−1^. Relative error in the association constants is 15%, error in binding enthalpies is 0.3 kcal mol^−1^.

We used isothermal titration calorimetry (ITC) to confirm the binding of tolcapone to WT-TTR (Fig. 2a). We also evaluated tolcapone binding to the FAC-associated V122I-TTR variant (Fig. 2b). Data analysis was performed considering two models: Model 1: Two identical and independent binding sites; Model 2: Two identical and cooperative binding sites^43^,^44^. The fit using model 2 was better for WT-TTR, whereas model 1 was better for V122I, according to parametric (F test) and non-parametric tests (Akaike's and Bayesian information criteria).

According to model 2, tolcapone binds to WT-TTR with Kd_1_=21 nM and Kd_2_=58 nM. In the case of tafamidis binding to WT-TTR, we obtained Kd_1_ and Kd_2_ values of 5.7 and 260 nM, respectively (Supplementary Fig. 3). These data are consistent with previously published experiments where Kd_1_ and Kd_2_ for tafamidis binding to WT-TTR were found to be 3.0–4.4 nM and 278–280 nM, respectively^29^,^45^. For tolcapone, the binding of the first ligand displays favourable binding enthalpy (Δ*h*_1_=−8.7 kcal mol^−1^) and binding entropy (*T*Δ*s*_1_=1.8 kcal mol^−1^; Fig. 2c). This first binding event is thus enthalpically driven, suggesting specific interactions between protein and ligand. The binding of tolcapone to the second site is almost entirely enthalpically driven (Δ*h*_2_=−9.7 kcal mol^−1^ and *T*Δ*s*_2_=0.2 kcal mol^−1^), which again suggests the formation of specific interactions.

According to model 1, tolcapone shows identical binding affinity for both V122I-TTR T_4_-binding sites, with a Kd=56 nM. The binding of tolcapone to V122I-TTR is entirely enthalpically driven with Δ*h*=−11.5 kcal mol^−1^ for each independent binding site and an unfavourable binding entropy *T*Δ*s*=1.6 kcal mol^−1^. From our ITC studies we estimate the Kd_2_ for binding of tafamidis to V122I-TTR to be 1.1 μM (Supplementary Fig. 3). These data are in agreement with previously published subunit exchange assays that estimate that the Kd_2_ of tafamidis for binding to V122I-TTR would be in the μM range^45^.

### Tolcapone stabilizes the TTR dimer–dimer interface

**Figure 3:**
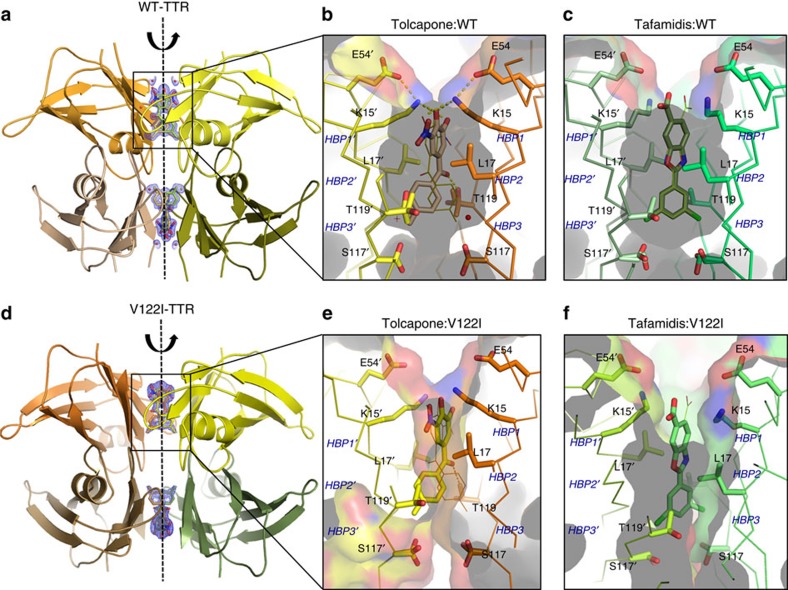
Crystal structures of WT-TTR/ligand and V122I-TTR/ligand complexes. (**a**) Global view of WT-TTR bound to tolcapone (ribbon representation). The electron density map of the two T_4_-binding sites of tolcapone are depicted. Broken line indicates the twofold symmetry axis of the dimer–dimer interface. Detailed and close up view of one of the WT-TTR T_4_-binding sites are shown in **b** for tolcapone (PDB: 4D7B) and **c** for tafamidis (PDB: 3TCT). (**d**) Global view of V122I-TTR bound to tolcapone (ribbon representation). The electron density map of the two T_4_-binding sites of tolcapone are depicted. Broken line indicates the twofold symmetry axis of the dimer–dimer interface. Detailed and close up view of one of the V122I-TTR T_4_-binding sites are shown in **e** for tolcapone (PDB: 5A6I), and **f** for tafamidis (PDB: 4HIS). Ligands and some of the TTR interacting residues are represented by sticks. Electron densities of the two structures are shown in Supplementary Fig. 5.

We obtained a co-crystal structure of tolcapone and WT-TTR at 1.15 Å (Fig. 3a,b, Supplementary Table 2 and Supplementary Fig. 4). This high-resolution crystal allows the unambiguous placement of tolcapone in the butterfly-shaped electron density map of the TTR dimer–dimer interface. The twofold symmetry axis along the hormone-binding pocket creates two binding modes of tolcapone related by a 180° rotation. The 4-methylphenyl ring of tolcapone sits deep within the inner cavity of the T_4_-binding site, establishing hydrophobic interactions with residues composing the two symmetrical T_4_-Halogen-Binding Pockets HBP2–2′ and HBP3–3′ (Ala108, Leu110, Ser117 and Thr119). In addition, a specific hydrogen bond interaction is formed between the central carbonyl group of tolcapone and the hydroxyl side chain of Thr119 (at 2.55 Å). The side-chains of Thr119 and Ser117 display two or three different conformations, respectively, probably caused by the interaction with the two symmetrical binding modes of tolcapone. On the outer binding region, the 3,4-dihydroxy-5-nitrophenyl ring of tolcapone is placed in the hydrophobic environment created by residues from the HBP2 and -2′ and HBP1 and -1′ pockets (Lys15, Leu17, Thr106 and Ala108). Remarkably, in this outer binding pocket the ɛ-amino group of Lys15 is sandwiched between the two hydroxyl groups of the phenyl ring of tolcapone (at 2.97 and 3.22 Å, respectively) and the carboxylate group of Glu54 (2.87 Å; Fig. 3b). These electrostatic interactions by Lys15 close the cavity around tolcapone, restricting the entrance of solvent into the HBP pockets (Supplementary Fig. 4) in such a way that the tolcapone salt bridge with Lys15 and the polar interactions with Thr119 are both partially shielded. The higher solvation interface between dimers in the TTR/tolcapone crystal structure with respect to the TTR/tafamidis structure (Supplementary Fig. 4) suggests a lower water rearrangement, contributing thus to tolcapone's lower desolvation entropy for binding.

We also obtained a co-crystal structure of tolcapone and V122I-TTR at 1.9 Å resolution (Fig. 3d,e and Supplementary Table 2). This is of particular relevance as the V122I mutation impacts directly the weaker dimer–dimer interface, resulting in an increased rate of tetramer dissociation^37^. As for the WT-TTR co-crystal, the twofold symmetry axis along the hormone-binding pocket creates two binding modes of tolcapone related by a 180° rotation. The conformation of tolcapone in the T_4_-binding site and the contacts it establishes with the mutant V122I protein are virtually identical to those found when it is bound to WT-TTR, explaining why tolcapone is also a high-affinity binder and a more effective aggregation inhibitor of the kinetically unstable, FAC-associated, V122I-TTR than tafamidis (Fig. 3f).

### Tolcapone selectively binds to TTR in human plasma

To become a drug for TTR-related diseases, tolcapone should selectively bind to TTR among the more than 4,000 human plasma proteins. To test this notion, tolcapone (10.8 μM final concentration) was added to human plasma and the samples were incubated for 24 h at 37 °C. The TTR concentration in plasma is in the 2.8–5.4 μM range; thus, sufficient ligand was present in the assay to saturate the two TTR-binding sites, assuming that the submicromolar ITC-derived Kds are also valid in this milieu. Plasma TTR was then immunoprecipitated and the stoichiometry of tolcapone relative to TTR in the precipitated fraction quantified by high-performance liquid chromatography. An assay using tafamidis was performed under the same conditions. The maximal binding stoichiometry possible is 2, because there are 2 T_4_-binding sites per TTR tetramer. The TTR-binding stoichiometry obtained for tolcapone in plasma is 0.87±0.09, whereas that of tafamidis is 1.12±0.09 (ligand/TTR), a value consistent with that previously reported^29^. These ratios represent a lower limit for the compound/TTR stoichiometry due to possible loss of bound compounds during sample processing. In any case, the data support tolcapone, as tafamidis, being highly selective for TTR in human plasma.

**Figure 4:**
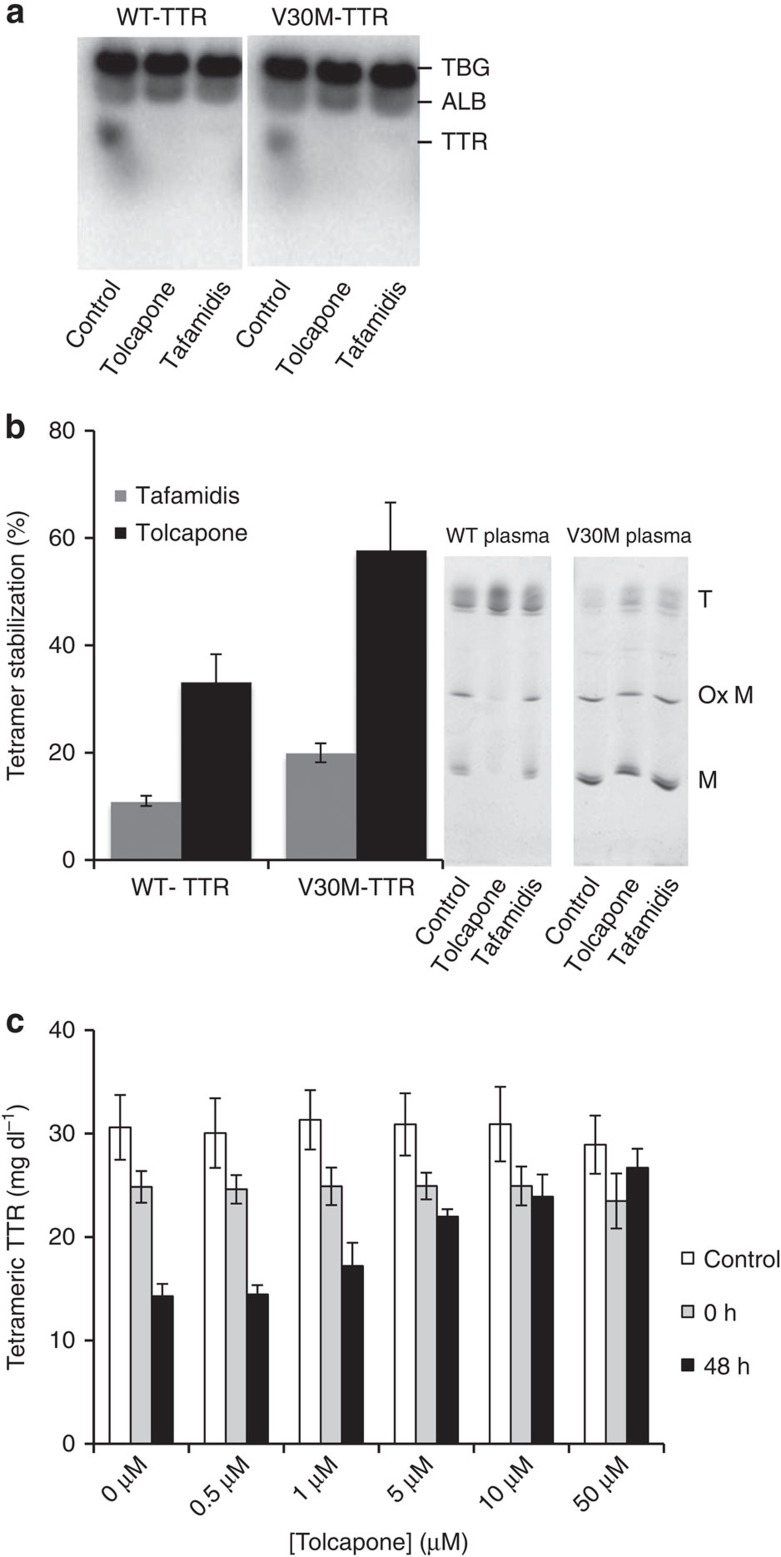
Tolcapone selectively binds and stabilizes WT and V30M-TTR in human plasma. (**a**) Human plasma of control or V30M-TTR carriers were incubated with [^125^I]T_4_ in the presence or absence of tolcapone or tafamidis (final concentration=1 mM). After 1 h at RT, the plasma proteins were separated by native gel electrophoresis. The gel was subjected to phosphor imaging and the radioactive bands were quantified. The band of TTR bound to radioactive T_4_ is not present in the plasma samples incubated with the small molecules, indicating an almost complete and selective displacement of T_4_ from the TTR-binding pocket by the compounds. Uncropped images of the films are shown in Supplementary Fig. 6. (**b**) Human plasma was incubated with 1.5 mM of tolcapone or tafamidis and tetramer stability assessed by IEF under semi-denaturing conditions^62^. The increase of the ratio between tetramers and other species in the gels is an indicator of TTR stabilization in drug-treated samples. Quantification of bands shows higher proportion of tetramers in the plasma samples treated with tafamidis and particularly in those treated with tolcapone. ‘T', ‘Ox M' and ‘M' indicate Tetramer, Oxidized Monomers and Monomers, respectively. Error bars indicate s.e.m. (*n*=3). Uncropped images of the gels are shown in Supplementary Fig. 7. (**c**) Human plasma samples were incubated with several concentrations of tolcapone for 15 min at RT. After incubation, an aliquot was separated for total TTR determination (control). Urea buffer was added and samples were incubated at RT for 0 or 48 h. After the indicated incubation times, the samples were crosslinked with glutaraldehyde as detailed in the experimental section, and the tetrameric TTR concentration determined by immunoturbidity. Addition of tolcapone to the plasma results in dose-response stabilization of tetrameric TTR. Error bars indicate s.e.m. (*n*=3).

We next tested the capacity of tolcapone and tafamidis to compete with T_4_ for TTR binding in human plasma from a normal (WT-TTR) and from an FAP patient carrying the V30M-TTR mutation. Plasma in the presence or absence of tolcapone and tafamidis was incubated with [^125^I]-labelled T_4_, followed by separation of T_4_-binding proteins using native gel electrophoresis and autoradiography (Fig. 4a)^42^. In both samples, in the absence of compounds, three main plasma proteins bind T_4_: T_4_-binding globulin (TBG), albumin (ALB) and TTR. In the presence of tolcapone and tafamidis, the radioactive T_4_-TTR band is no longer visible, indicating that these molecules can selectively compete with T_4_ for TTR binding in plasma.

### Tolcapone stabilizes WT-TTR and V30M-TTR in human plasma

The ability of tolcapone to prevent TTR tetramer dissociation in the plasma of normal and FAP V30M patients was monitored by isoelectric focusing (IEF) electrophoresis under semi-denaturing conditions (4 M urea). Tafamidis was also used as control. Plasma samples were incubated with 1.4 mM solution of test compounds overnight at 4 °C followed by 1 h incubation at RT. Samples were processed as detailed in the experimental section^42^. A representative gel is shown in Fig. 4b. The presence of TTR monomer (M), oxidized monomer (Ox M) and several bands of lower pI corresponding to tetramers can be discriminated. The extent of tetramer stabilization was calculated as the ratio of tetramer over total TTR in the presence of compounds, relative to the same value in their absence. Tafamidis and tolcapone stabilized WT and V30M-TTR tetramers in plasma, with tolcapone displaying a higher stabilizing activity (Fig. 4b).

Stabilization of plasma TTR under physiological conditions was confirmed using an immunoturbidity assay^29^ (Fig. 4c). A pool of human plasma from healthy volunteers was incubated with different concentrations of tolcapone before denaturation with 4.8 M urea during 48 h. After urea treatment, samples were crosslinked with glutaraldehyde and the concentration of TTR determined by immunoturbidity. Treatment with urea and glutaraldehyde decreases immunoturbidity signal due to the extensive modification of denatured TTR monomers by glutaraldehyde, hampering antibody recognition^33^. As TTR denaturation is preceded by tetramer dissociation, the signal roughly correlates with tetramer stability. Preincubation with tolcapone stabilized TTR in a dose-responsive manner, conferring resistance to urea denaturation and preventing the loss of signal after 48 h of urea treatment (Fig. 4c).

### Tolcapone prevents TTR-induced cytotoxicity in cell culture

**Figure 5:**
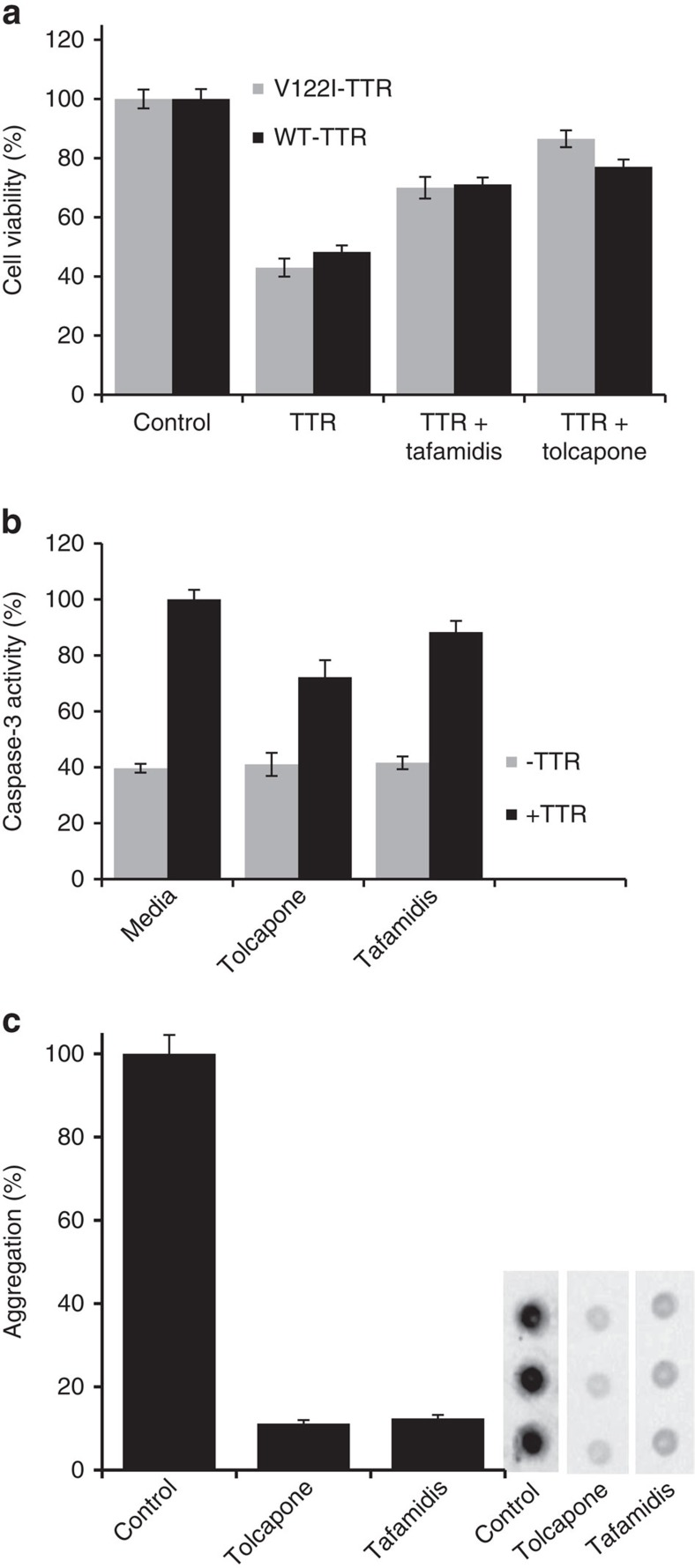
Tolcapone prevents TTR-induced cytotoxicity of TTR variants and inhibits L55P-TTR aggregation under physiological conditions. (**a**) Tolcapone protects human cardiomyocytes from the toxic effect of V122I-TTR and WT-TTR. AC16 cells were incubated for 24 h with WT or V122I-TTR in the presence or absence of 2 molar equivalents of tolcapone or tafamidis. Resazurin reduction assay was used to measure cell viability. Percentage of cell viability was calculated compared with cells treated with vehicle only. Both compounds rescued the cells from the toxicity induced by TTR. Error bars indicate s.e.m. (*n*=8). (**b**) Tolcapone lowers cytotoxicity of Y78F-TTR aggregates. RN22 cells were exposed to aggregates generated in the absence or presence of tolcapone or tafamidis for 24 h. Caspase-3 activation on cell lysates was used to probe cytotoxicity. Both compounds lowered caspase-3 activation promoted by the aggregates. Error bars indicate s.e.m. (*n*=4). (**c**) Tolcapone prevents aggregation of L55P-TTR under physiological conditions. RN22 cells expressing L55P-TTR under the control of methallothienin promoter were incubated in the presence or absence of tolcapone or tafamidis (1 μM). Secreted L55P-TTR aggregation was monitored by a filtration assay followed by a dot blot (representative blot triplicates are shown on the right panel). Uncropped images of the blots are shown in Supplementary Fig. 8. Filtration of the medium retains high molecular mass aggregates that are detected using a rabbit anti-human TTR antibody. Intensities of the dots were measured, percentage of aggregation was calculated with respect to cells treated with vehicle, and plotted as bars (left panel). Error bars indicate s.e.m. (*n*=3).

To test the potential therapeutic activity of tolcapone for the cardiac forms of TTR amyloidoses, we used an established cell model system based on the use of AC16 cells^46^, a human cardiac cell line derived from adult cardiomyocytes of the ventricle, the site of TTR deposition in FAC and SSA^47^,^48^. AC16 cells were treated with 8 μM of cytotoxic WT-TTR and V122I-TTR incubated in the presence or absence of tafamidis or tolcapone (16 μM; i.e., two molar equivalents with respect to TTR). Cell treatment with WT-TTR and V122I-TTR alone caused a significant reduction in cell viability measured by resazurin reduction assay (∼60%). Both tafamidis and tolcapone rescued this toxic effect, with tolcapone exhibiting a higher protective activity (Fig. 5a).

We also analysed whether the TTR anti-aggregational activity of tolcapone results in a reduction of the toxicity associated with the formation of Y78F-TTR oligomers in a FAP cell culture model^49^. Y78F-TTR was incubated in the absence or presence of the selected compounds at 37 °C for 6 days to generate cytotoxic oligomers. Rat Schwannoma cells (RN22) were exposed to these aggregates (2 μM) for 24 h and cell lysates were used to determine Caspase-3 activation levels. The addition of TTR aggregates in the absence of compounds increased the intracellular levels of Caspase-3 compared with control cells (Fig. 5b). Incubation with tolcapone and tafamidis promoted 27.8%±6.1 and 11.6%±4.0 reduction in Caspase-3 activation, respectively (Fig. 5b).

To determine whether tolcapone can inhibit extracellular TTR fibrillogenesis under physiological conditions, we used RN22 cells stably transfected with the highly pathogenic FAP-associated L55P-TTR variant^50^ under the control of the metallothionein promoter^51^. Upon induction with ZnSO_4_, cells secreted L55P-TTR to the extracellular milieu. RN22 cells were incubated with 1 μM of tolcapone or tafamidis in the medium for 48 h, the expression of the recombinant protein was induced by addition of 100 μM ZnSO_4_ and the incubation continued for 24 h. The extent of extracellular L55P-TTR aggregation was monitored by a filter trap assay followed by TTR immunodetection. Both compounds exhibited strong effect, reducing ∼90% L55P-TTR aggregation compared with untreated cells (Fig. 5c).

### Tolcapone binds to and stabilizes TTR in transgenic mice

**Figure 6:**
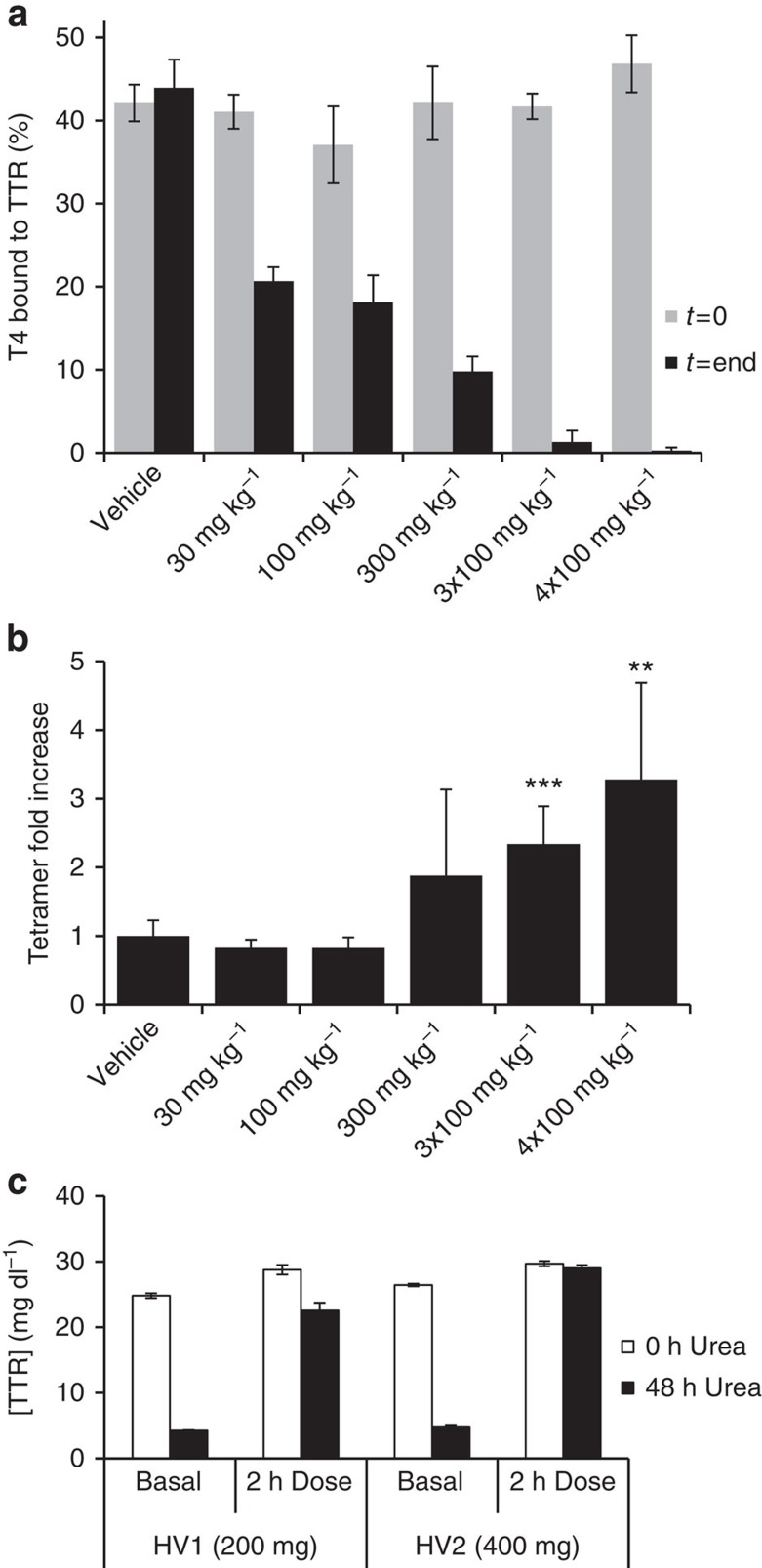
Tolcapone is orally available and stabilizes TTR in humans and in transgenic mice expressing the amyloidogenic human V30M-TTR variant. (**a**) T_4_ competition was assessed by gel electrophoresis of plasma samples from the transgenic mice expressing human V30M-TTR, before and after oral administration of tolcapone (*t*=0 and *t*=end, respectively). Plasma samples were treated with radiolabelled T_4_ and subjected to native PAGE. The fraction of radiolabelled T_4_ bound to the band corresponding to TTR is represented. Error bars indicate s.e.m. (*n*=4). (**b**) TTR tetramer stability in the plasma of control and tolcapone-treated transgenic mice was assessed by IEF under semi-denaturing conditions^62^. The ratio between the TTR tetramers and the rest of the TTR species in the IEF gels was calculated for controls and tolcapone-treated mice. The data are expressed as fold increase of the ratio TTR tetramer/total TTR with respect to samples from vehicle-treated animals. ***P<0.001 and **P<0.01 (Student's *t*-test). Error bars indicate s.e.m. (*n*=3). (**c**) Tetrameric TTR stabilization in plasma from humans treated with an oral dose of 200 or 400 mg of tolcapone (subjects HV1 and HV2, respectively). TTR stabilization was quantified by immunoturbidity after urea denaturation with subsequent crosslinking with glutaraldehyde as detailed in the experimental section. Basal, indicates plasma obtained before tolcapone administration. White bars correspond to plasma samples crosslinked 0 h after urea addition, black bars correspond to plasma samples crosslinked after 48 h of urea denaturation. Bars correspond to average TTR tetramer concentration of three independent determinations, error bars represent standard deviation (*n*=3).

We addressed the effects of tolcapone in transgenic mice expressing the FAP-associated human V30M-TTR variant^52^. A dose of tolcapone ranging from 30 to 300 mg kg^−1^ was administered by gavage, followed by an identical dose after 8 h; alternatively the rodents were treated with three or four consecutive doses of 100 mg kg^−1^ spaced every 3 h. The animals were killed 1 h after the last dose. Plasma samples were obtained before dosing (*t*=0) and at the end of the experiment. T_4_ competition assays by native gel electrophoresis were performed in plasma following the same protocol as in Fig. 4a. At basal conditions (*t*=0), around 40% of the total radioactivity was associated with the TTR band in samples from all animals. At the end of the treatment, the amount of T_4_ remaining bound to TTR decreased in a dose-dependent manner (Fig. 6a), indicating that tolcapone binds to the TTR T_4_-binding sites *in vivo*.

The ability of tolcapone to stabilize V30M-TTR in the plasma of animals was analysed under semi-denaturing conditions by isoelectric focusing as in Fig. 4b. The tetramer/total TTR ratio in the plasma of tolcapone-treated animals was higher than in those treated with vehicle alone (Fig. 6b), indicating that the binding of the compound to TTR results in a significant stabilization of the native V30M-TTR state *in vivo*.

### Orally administered tolcapone stabilizes TTR in humans

TTR stabilization by tolcapone was also confirmed in humans, using plasma samples from two healthy volunteers treated with a commercial oral formulation of tolcapone. One volunteer (HV1) was treated with a dose of 200 mg of tolcapone, a usual dose for Parkinson's treatment, and the other (HV2) with 400 mg administered as two sequential doses of 200 mg, separated by a 30-min interval. Plasma samples were obtained 30 min before (basal) and 2 h after the last administration, which is the time at which peak plasma concentration occurs (Tmax)^53^. The concentration of tolcapone in all plasma samples was measured using an ultra-performance liquid chromatographic (UPLC)-MS/MS analytical method (see Methods for details; Table 1). TTR stabilization *in vivo* was measured using an immunoturbidimetric assay as in Fig. 4c. The fraction of initial tetramer concentration (FOI) was determined in triplicate for each sample (FOI=TTR tetramer after 48 h urea treatment/TTR tetramer at time 0 in the presence of urea).

As shown in Fig. 6c, oral treatment with tolcapone stabilizes TTR, preventing its denaturation. In the absence of tolcapone (basal), there was a significant loss of tetrameric TTR signal after urea treatment (compare 0 h urea and 48 h urea bars in basal samples). The FOI in basal samples of subjects HV1 and HV2 were 0.17±0.00 and 0.18±0.01, respectively. In contrast, after treatment with tolcapone, the loss of TTR tetramer signal after 48 h of urea treatment is very low, with FOI values of 0.78±0.04 and 0.98±0.02 in HV1 and HV2, respectively, achieving in the later an almost complete protection against urea-induced TTR tetramer denaturation. These data are consistent with the fact that the measured tolcapone levels in HV2, who received two doses of tolcapone, were higher than those found in HV1 (Table 1).

Altogether these results indicate that oral administration of tolcapone in humans effectively stabilizes native tetrameric TTR. According to the Vyndaqel European Public Assessment Report (EMA/815723/2011), following a single dose of 120 mg tafamidis, the average concentration of tafamidis in plasma at Tmax was 14.4 μM, which resulted in an average percent stabilization of TTR of 189%. For tolcapone, after a single dose of 200 mg, its concentration in plasma at Tmax was 13.6 μM, resulting in 359% TTR stabilization; these data indicate that at similar Tmax concentrations, tolcapone TTR stabilization capacity is 1.9-fold higher than that of tafamidis.

## Discussion

According to the National Institutes of Health, the average time between the discovery of a new drug candidate and its market approval is 13 years—a process with an associated failure rate higher than 95%, and an average cost of $1,778 million^54^. Repurposing FDA-approved drugs for new indications can offer an accelerated pathway for new treatments because of faster early phases of clinical development, shortened drug optimization timelines and reduced failure rates due to pharmacokinetic and safety issues. Therefore, one of the National Institutes of Health missions consists in repurposing known drugs for new indications.

Diflunisal, the only FDA-approved drug that has already shown to inhibit FAP progression in clinical trials^35^ is a promising molecule to preserve the quality of life in patients affected by hereditary TTR amyloidosis. However, diflunisal can produce significant gastrointestinal and cardiovascular side effects and thus, it may not be appropriate for chronic administration to a large subpopulation of patients, such as those with high blood pressure, or with already compromised gastrointestinal systems^55^,^56^. Therefore, additional alternative, potent and safe small molecules are still needed to effectively treat the TTR amyloidoses.

We describe here how the application of a drug repositioning protocol for the TTR amyloidoses has crystallized in the discovery of tolcapone, a promising FDA-approved drug for the treatment of SSA, FAP, FAC and uniquely, TTR leptopmeningeal amyloidosis.

Tolcapone occupies the T_4_-binding sites located at the TTR dimer–dimer interface, being able to dock into these pockets with notable selectivity in the highly protein-crowded human plasma environment. The binding of tolcapone to the TTR T_4_ pockets is not expected to result in thyroid metabolic defects because the contribution of TTR to plasma T_4_ transport is low, relative to that of TBG and ALB^10^,^57^.

Tolcapone binding to the TTR's T_4_ pockets prevents amyloidogenesis by acting on the protein native state, raising the energy barrier for tetramer dissociation, which is the rate-limiting step of the TTR amyloid formation cascade^15^,^41^. This mode of action is nowadays the preferred therapeutic approach for TTR amyloidosis treatment as it appears that soluble oligomeric species, presumably amyloid fibril precursors, exert the main cytotoxic effects in tissues, well before there is massive protein deposition^58^,^59^.

*In vitro*, tolcapone binds with high affinity and strongly inhibits the aggregation of WT-TTR as well as the kinetically unstable V122I-TTR variant, involved in cardiac TTR amyloidosis. It also inhibits the aggregation of the leptomeningeal-associated A25T-TTR variant. Importantly, tolcapone is significantly more potent than tafamidis at preventing TTR aggregation, independently of the TTR variant, concentration and assay used for its evaluation.

Tolcapone's stabilizing effect rescues WT- and V122I-TTR-induced cardiotoxicity in a well-characterized cell culture model system for FAC^47^,^48^. In the absence of a transgenic mouse model expressing the human V122I-TTR variant, this cardiac tissue culture system using adult cardiomyocytes of the ventricle, the site of TTR deposition, is the most pathophysyologically relevant system for V122I-TTR cardiac amyloidosis^46^. Moreover, tolcapone partially prevents caspase-3 activation induced by Y78F-TTR oligomers in the RN22 cellular model for FAP^49^. In both the FAC and the FAP cellular model systems, the protective activity of tolcapone against TTR proteotoxicity was higher than that of tafamidis.

Tolcapone binds to and kinetically stabilizes tetrameric TTR *ex-vivo* in human plasma from WT-TTR individuals and from carriers of V30M-TTR, the most common FAP-associated TTR variant worldwide^60^. It also stabilizes human V30M-TTR *in vivo* in plasma from transgenic mice, after oral administration of the drug. Importantly, tolcapone prevents native WT-TTR dissociation and amyloidogenesis *in vivo* in human subjects.

The co-crystal structures obtained for WT-TTR and V122I-TTR with tolcapone indicate that the small-molecule interacts with both the inner cavity and the periphery of the T_4_-binding site. The binding of tolcapone involves more polar contacts as well as hydrophobic interactions than in the case of tafamidis. The formation of a specific hydrogen bond with Thr119 and Thr119′ and a salt bridge with Lys15 and Lys15′ at the dimer–dimer interface might explain why the binding of tolcapone to the T_4_ sites is essentially enthalpically driven. This is particularly true for binding to V122I-TTR. Indeed, V122 is located on the periphery of the H β-strand, which forms an antiparallel β-sheet with another monomer, stabilizing the AC/BD dimer interface. The V122I mutation impacts directly on the stability of this interface and tolcapone compensates this destabilizing effect by bridging the H β-strands of adjacent monomers through specific hydrogen bonds and ionic interactions. Although binding enthalpy provides a drug candidate with affinity and selectivity, enthalpic optimization may take years and often only appear in second-generation products^61^. In tolcapone, this property appears to be already optimized for binding to both T_4_ pockets where multiple interactions with different subunits of the protein occur.

In summary, we have shown that tolcapone, a FDA-approved drug, is a potent inhibitor of TTR amyloidogenesis, prevents early events of WT and mutant TTR-induced cytotoxicity, binds to and stabilizes TTR in human plasma *ex-vivo*, to human V30M TTR in transgenic mice plasma *in vivo* and importantly, to human plasma TTR *in vivo* after oral administration at the therapeutic doses used for Parkinson's disease treatment.

At similar Tmax concentrations (∼14 μM) tolcapone's TTR stabilizing effect *in vivo* is almost two times larger than that of tafamidis, in good agreement with all our *in vitro* data. In contrast, diflunisal attains a high concentration in serum after oral administration (>140 μM)^33^, which compensates for its low affinity and negative binding cooperativity^19^ (Kd_1_=75 nM; Kd_2_=1100, nM). In any case, the ratio between the maximum concentration attained in plasma when the drug is administered at therapeutic doses and the one needed to theoretically saturate the two T_4_-binding sites in WT-TTR is 1.8- and 4.5-fold higher in tolcapone than in diflunisal and tafamidis, respectively.

The efficacy of a given drug depends on its target affinity, specificity and attainable serum concentrations, but also on its clearance rate. A limitation of tolcapone in relation to both diflunisal and tafamidis is its shorter half-life in plasma (∼2–3 h); however, preliminary data indicate that this limitation can be at least partially overcome using slow release formulations.

Tolcapone has the potential of becoming an efficacious small molecule to prevent the onset of TTR deposition diseases; tolcapone could be in the market in a relatively short time frame as it has already entered into clinical trials (ClinicalTrials.gov Identifier: NCT02191826/EudraCT number: 2014-001586-27). Importantly, the unique ability of tolcapone to penetrate the blood–brain barrier^40^ and to reduce A25T-TTR aggregation indicate that it could become the first pharmacologic treatment available for TTR leptomeningeal amyloidosis.

## Methods

### Recombinant TTR expression and purification

WT, V30M, Y78F, A25T and V122I-TTR variants were prepared as in refs 36, 38.The recombinant TTR proteins were produced using a pET expression system (Novagen). The mutant proteins were prepared by PCR site-directed mutagenesis using the QuickChange kit (Stratagene). Both WT and mutant proteins were expressed in *Escherichia coli* BL21- (DE3) cells harbouring the corresponding plasmid. Expression cultures in lysogeny broth (LB) medium containing 50 μg ml^−1^ kanamycin were grown at 37 °C to an optical density (at 600 nm) of 0.6, then induced by addition of isopropyl-β-D-thiogalactoside (1 mM final concentration), grown at 37 °C for 20 h, and harvested by centrifugation (13,700*g* for 15 min). After cell lysis by sonication, intra-cellular proteins were fractionated by ammonium sulfate precipitation. The TTR-containing fraction precipitated between 55 and 85% ammonium sulfate. The precipitate was dissolved in 20 mM Tris, pH 7.2, 0.1 M NaCl and dialysed against the same buffer. It was applied to a Q-Sepharose High Performance (Amersham Biosciences) anion exchange column and eluted with a linear gradient 0.1–0.5 M NaCl in 20 mM Tris, pH 7.2. TTR-enriched fractions were dialysed against 5 mM Tris, pH 7.2, 2.5 mM NaCl, lyophilized and redisolved in a small volume of buffer (10 mM Tris, pH 7.2, 0.1 mM NaCl). The protein was further purified by gel filtration chromatography on a Superdex 75 prep grade column (Amersham Biosciences) and eluted with 10 mM Tris, pH 7.2, 0.1 M NaCl. Purest fractions were combined and dialysed against 20 mM phosphate buffer, pH 7.6, 100 mM KCl and stored at 4 °C. The purity of protein preparations was >95% as judged by SDS–PAGE. Protein concentration was determined spectrophotometrically at 280 nm using an extinction coefficient of 77,600 M^−1^ cm^−1^.

### TTR *in vitro* aggregation inhibition assays

The initial screening protocol used for the 30 top ranked molecules from the docking experiment is described in detail elsewhere^36^. Briefly, Y78F-TTR solutions (7 μM in 10 mM sodium phosphate, 100 mM KCl, 1 mM EDTA, pH 7.6) were incubated with varying concentrations of the test compounds (ranging from 0 to 40 μM) for 30 min at 37 °C. All compounds stock solutions were prepared in dimethylsulphoxide (DMSO). DMSO concentration was adjusted to 5% (v/v) in the final reaction assay mixture. After this incubation period, the pH of the solutions was lowered by dilution with 400 mM sodium acetate, 100 mM KCl, pH 4.2. TTR aggregation was followed by turbidity at 340 nm for 1.5 h on a 96-well microplate reader. All assays were performed at least in duplicate. Aggregation of TTR was considered maximum in the absence of compounds (100% aggregation). The same assay was performed for WT-TTR and the V122I-TTR variant except that turbidity at 340 nm was measured after 72 h incubation at 37 °C. For A25T-TTR, incubation was measured after 22 h incubation at pH 5.0 and 37 °C, as previously described^8^. Because tolcapone exhibits dose-dependent absorbance at 340 nm (Supplementary Fig. 2), each individual measurement was corrected with a buffer containing the same concentration of compound but devoid of TTR.

### Sedimentation assay

TTR aggregation can also be measured by sedimentation using centrifugation. It has previously been demonstrated that the TTR can be partitioned between precipitates and supernatant with a recovery rate of >90% with respect to the total initial protein^48^. TTR aggregation induction was performed as described above. The samples of WT, V122I-TTR and A25T-TTR incubated in the presence of different concentrations of tolcapone were centrifuged at 20,000*g* for 1 h. The supernatant was carefully removed, and 400 μl of Guanidine (8 M) was added to each sample and subsequently incubated for 1 h to unfold the aggregated TTR. The amount of resolubilized TTR was measured by absorbance at 280 nm. The percentage of TTR precipitated was calculated with respect to total (soluble) TTR at time 0.

### TTR stabilization by tolcapone in the presence of urea

WT-TTR (1 μM in PBS) was incubated with or without 10 μM of tolcapone for 30 min. Urea was added to attain a range of final concentrations (0–9 M) and the samples were incubated for 72 h at RT. Fluorescence intensity measurements were performed on a Jasco FP-8200 Spectrofluorometer. Trp fluorescence was obtained by exciting the samples at 280 nm and emission was collected from 300 to 400 nm. The fluorescence intensity ratio 355/335 nm was plotted against urea concentration as a sensor of folded protein, which for TTR in urea is equivalent to tetramer integrity^15^. In another setting, WT-TTR (1.8 μM in PBS) was incubated with several concentrations of tolcapone (0.9–8 μM) in 6.5 M urea for 72 h at RT. The fraction of unfolded TTR was measured and calculated as above. The TTR fluorescence after incubation at RT for 72 h in 9 M urea was considered to correspond to that of the unfolded state. Incubation of WT-TTR for 48 h with Guanidine hydrochloride (6.5 M) resulted in the same extent of denaturation. As a control, we also excited 1 and 10 μM of tolcapone (PBS) in the absence and presence of TTR and observed that this compound has no fluorescence and does not interfere with Trp excitation or emission at the used wavelengths (Supplementary Fig. 2).

### Isothermal titration calorimetry

ITC was used to study the interaction between TTR and tolcapone or tafamidis using an Auto-iTC200 calorimeter (MicroCal). TTR at 5 μM located in the calorimetric cell was titrated against the drugs at 100 μM in the injection syringe in PBS buffer pH 7.0, 100 mM KCl, 1 mM EDTA, 2.5% DMSO, at 25 °C. A stirring speed of 1,000 r.p.m. and 2 μl injections were programmed, with consecutive injections separated by 150 s to allow the calorimetric signal (thermal power) to return to baseline. Experimental data were analysed with a general model for a protein with two ligand-binding sites^43^,^44^ implemented in Origin 7.0 (OriginLab).

### Crystallography and structure determination

Co-crystals of WT-TTR/tolcapone and of V122I-TTR/tolcapone were obtained at 18 °C by sitting drop vapour diffusion methods after mixing a tenfold molar excess of ligand with protein to ensure saturation. The reservoir solution contained between 15 and 25% PEG 400, 200 mM calcium chloride, 100 mM HEPES, pH 7.0. Single crystals appeared after 3 days from equal volumes of protein solution (10 mg ml^−1^ in 50 mM Tris-HCl (pH 8.0), 100 mM KCl, 1 mM EDTA) and reservoir solution. Crystals were cryo-protected in reservoir buffer containing 10% glycerol and flash-frozen in liquid nitrogen before diffraction analysis. Diffraction data were recorded from cryo-cooled crystals (100 K) at the ALBA synchrotron in Barcelona (BL13-XALOC beamline). Data were integrated and merged using XDS and scaled, reduced and further analysed using CCP4 (Supplementary Table 2). The structure of TTR/tolcapone complex was determined from the X-ray data by molecular replacement using a previous TTR structure (PDB code 3TCT) as a model using the programme MolRep. The initial electron density maps showed the butterfly electron density map corresponding to tolcapone in the TTR dimer–dimer interface that was manually built using the programme COOT. Model refinement was performed with Refmac and Phenix. Ramachandran analysis shows 98.00% of residues (196) are in preferred regions and only 2.00% of residues (4) are in allowed regions for the WT-TTR/tolcapone structure. For the V122I-TTR/tolcapone structure, 96.30% of residues (104) are in preferred regions and only 3.70% of residues (4) are in allowed regions. Refinement and data statistics are provided in Supplementary Table 2. Structural representations were prepared with PyMOL.

### Binding competition assays using radioactive T_4_

Displacement of T_4_ from TTR in plasma (*ex vivo*) was assayed qualitatively by incubation of 5 μl of human plasma with 0.25–0.5 μl of [125I]T_4_ (specific radioactivity 1,250 μCi μg^−1^; concentration 320 μCi ml^−1^; Perkin Elmer) in the presence of 1 mM of test compounds. The negative control was performed by adding 1 μl of PBS. The samples were incubated for 1 h at RT. The same incubations were performed using mouse plasma treated *in vivo* with the small molecules or vehicle control (gavage administration). After incubation, plasma proteins were separated by native PAGE as in ref. 42. The gels were dried and subjected to phosphor imaging (Typhoon 8600; Molecular Diagnostics, Amersham Biosciences) for quantification, and the bands analysed using the ImageQuant program version 5.1. The three observed bands correspond to the three-major T_4_-binding plasma proteins: TBG, ALB and TTR, the later presenting the most anodal migration.

### Stability studies by native PAGE and isoelectric focusing (IEF)

TTR tetramer stabilization by compounds was assessed by IEF under semi-denaturing conditions as described in detail in ref. 62. Briefly, 30 μl of human plasma from WT and V30M-TTR carriers (*n*=6) were incubated overnight at 4 °C with 5 μl of compounds at 10 mM (in DMSO). Plasma samples from transgenic mice expressing V30M-TTR that had been treated with tolcapone or vehicle were analysed in the same way (see below). The plasma samples were subjected to native PAGE and the gel band containing TTR was excised and applied to an IEF gel. IEF was carried out under semi-denaturing conditions (4 M urea), containing 5% (v/v) ampholytes, pH 4–6.5 (GE Healthcare), at 1,200 V for 6 h. Proteins were stained with Coomassie blue. The gels were scanned (HP Scanjet 4470c, Hewlett Packard) and subjected to densitometry analysis using the ImageQuant program. The ratio of TTR tetramer over total TTR (TTR tetramer+monomer) was calculated for each plasma sample and the % TTR tetramer stabilization was calculated as 100 × ((ratio sample—ratio non-treated)/ratio non-treated). Where ‘ratio sample' is tetramer/total TTR in the presence of compound; and ‘ratio non-treated' is tetramer/total TTR of non-treated plasma sample from the same donor. For the mice plasma analysis, each gel contained at least one sample from control animals serving as internal control, to correct for the gel to gel variability (seven gels were run in total, each sample was analysed at least in two independent gels). The ratios of TTR tetramer/Total TTR were calculated for each sample collected at the end of the assay, from controls and drug-treated mice. The ratio of each sample was then expressed as fold increase with respect to control samples from vehicle-treated animals (ratio drug treated sample/average ratio vehicle samples from the corresponding gel). The results are represented as average fold increase±standard deviation.

### *Ex vivo* stability studies by immunoturbidity

A pool of lithium-heparin plasma samples from healthy volunteers stored at −80 °C was thawed on ice. Partially clotted particles were removed by spinning the samples for 2 min at 10,000*g*. Samples were then incubated with various concentrations of tolcapone (from a DMSO stock stored at −20 °C), for 15 min at RT. Addition volume was limited to maximum 1.5% (v/v) of sample. A control in which DMSO vehicle was added was included in the experimental design. Samples were then mixed with a urea solution (8 M in 50 mM potassium phosphate pH 7.0, 100 mM NaCl, 1 mM dithiothreitol, 1 mM EDTA) to a final urea concentration of 4.8 M, and incubated for various time points at controlled RT conditions. At the time of measuring, an aliquot was taken and a 2.5 M glutaraldehyde solution was added to a final concentration of 40 mM. The samples were quickly vortexed and incubated for 4 min at RT. Then, NaBH_4_ was added to a final concentration of 48 mM to quench the reaction. Samples were transferred to a measuring tube and read by immunoturbidity after 5 min at RT. Immunoturbidity was measured in a Siemens Automate Dimension EXL200 instrument, using the Siemens Prealbumin (PALB) reagent and calibrator according to the manufacturer's instructions. The slight decrease observed in samples crosslinked immediately after adding urea (time 0) possibly reflects changes in antibody recognition not directly associated with TTR denaturation, as they are not affected by the presence of compound. Experiments were performed at least three times in duplicate.

### Cell toxicity assays

The AC16 human cardiomyocyte cell line^46^ was maintained in DMEM:F12 supplemented with 10% fetal bovine serum (FBS) 2 mM L-glutamine, 100 U ml^−1^ penicillin, 100 μg ml^−1^ streptomycin at 37 °C in a 5% CO_2_ incubator as in refs 38, 47. AC16 cells (70–90% confluent) were seeded in black wall clear bottom 96-well plates (250 cells per well) in Opti-MEM, supplemented with 5% FBS, 2 mM L-glutamine, 100 U ml^−1^ penicillin, 100 μg ml^−1^ streptomycin, 1 mM HEPES and 45 mM CaCl_2_ (seeding Opti-MEM medium) and incubated overnight at 37 °C. The seeding Opti-MEM medium was removed and immediately replaced with 100 μl per well of the appropriate TTR solutions or vehicle (1:1) HBSS/Opti-MEM supplemented with glutamine and antibiotics and 0.4 mg ml^−1^ fatty acid- free BSA. AC16 cells were treated with TTR/compound mixtures (1:2 molar equivalents, final TTR concentration 8 μM), TTR alone (8 μM), drugs alone (16 μM) or vehicle controls, for 24 h at 37 °C. Cell viability was then measured by resazurin reduction assay, a process by which metabolically active cells reduce the substrate resazurin into the highly fluorescent resorufin^47^. Cell viability was calculated as percentage of fluorescence intensity (Exc/Em 530/590 nm) of the treated cells with respect to cells treated with vehicle only (no TTR or compounds) after subtraction of the blanks (wells with no cells). The assays were done at least in triplicate and repeated twice. The data shown represent the average of two independent experiments, the bars represent the standard error of the mean (s.e.m.).

Rat Schwannoma cells (RN22, American Type Cell Collection), 80% confluent cells in DMEM with 10% FBS, were exposed for 24 h to 2 μM of Y78F-TTR oligomers as detailed in ref. 36. These oligomers were obtained by incubation of soluble Y78F-TTR either in the absence or in the presence of 10 M equivalent excess (final concentration 20 μM) of test compounds at 37 °C for 6 days. The cells were then trypsinized and cell lysates were prepared and used for determination of caspase-3 activation with the CaspACE fluorimetric 96-well plate assay system (Sigma), following the manufacturer's instructions. Protein concentration in the cell lysates was determined with the Bio-Rad Bradford protein assay kit (Bio-Rad). Caspase-3 activation values were normalized by protein concentration in each experimental condition. The percentage of caspase-3 activation relative to the maximal activation was calculated. The maximal caspase-3 activation was considered to be that of cells treated with oligomers in the absence of inhibitors (media+TTR oligo). The assays were done in duplicate and repeated twice. The data shown represent the average of two independent experiments, the bars represent the standard error of the mean (s.e.m.). The data were analysed by Student's *t*-test using Microsoft Excel to establish significant changes.

### Dot-blot filter assay for quantification of TTR aggregation

The rat Schwannoma cell line RN22 stably co-transfected with TTR L55P cDNA under the control of the metallothionein promoter was used to test the inhibitors of TTR aggregation as in ref. 51. Cells were grown in six-well cell culture plates to 50% confluence in DMEM supplemented with 10% FBS, 1% penicillin/streptomycin and G418 (1 mg ml^−1^, Calbiochem) at 37 °C in a 95% humidified atmosphere and 5% CO_2_. To investigate the effect of test compounds on TTR aggregation, cells were incubated with 1 μM of tolcapone and tafamidis in the medium for 48 h and then for further 3 days with the compounds simultaneously with 100 μM ZnSO_4_ (to induce TTR expression), until reaching ∼80% cell confluence. The cells were then incubated with serum-free medium supplemented with ZnSO_4_ and test compounds for additional 24 h. TTR in the medium was quantified by ELISA, and the volume of medium corresponding to 500 ng of TTR was applied on to a 0.2-μm pore cellulose acetate membrane filter (Schleicher and Schuell) using a manifold system (Gibco BRL) to separate soluble proteins from aggregates. TTR was immuno-detected using a rabbit anti-human TTR (DAKO, A0002; 1:500) followed by a secondary antibody, anti-rabbit IgG-horseradish peroxidase conjugate (1:1,500). Detection was performed with ECL (GE Healthcare). Dot blot quantification was performed using the ImageQuant program. The percentage of aggregation inhibition was calculated as 100 × ((Control—Treated)/Control). Experiments were repeated three times in triplicate.

### Tolcapone oral administration in mice

Animal experiments were approved by the Portuguese General Veterinarian Board and animals were kept and used strictly in accordance with the National rules and the European Community Council Directive (86/609/EEC) for the care and handling of laboratory animals. Transgenic mice expressing human V30M-TTR received oral doses of tolcapone by gavage as follows: (I) 30 mg kg^−1^, two doses spaced 8 h (six animals); (II) 100 mg kg^−1^, two doses spaced 8 h (six animals); (III) 300 mg kg^−1^, two doses spaced 8 h (six animals); (IV) 100 mg kg^−1^, three doses in 3 h intervals (three animals) and (V) 100 mg kg^−1^, four doses in 3 h intervals (three animals). Nine animals administered with vehicle (PBS with 0.04% Tween 80 and 0.2% methylcellulose) were used as controls. Blood was collected from tail vein 16 h before treatment with the first dose (time, *t*=0), to minimize the stress of the animals before gavage. Collection of blood at the end of the experiment (*t*=end) was done from the posterior vena cava upon killing the animals after anaesthesia with ketamine/medetomide (75 and 1 mg kg^−1^, respectively). The animals were killed 1 h after the administration of last dose, which corresponds to 9 h for groups I–III and vehicle; 7 h for group IV and 10 h for group V.

### Tolcapone oral administration in humans

Two healthy volunteers of 49 years of age were treated with tolcapone (TASMAR) with a single dose of 200 or two doses of 200 mg in a 30-min interval. Blood was collected 30 min before administration and 2 h after the dose in BD Vacutainer tubes with Lithium-Heparin and gel for plasma separation, following the manufacturer's instructions. Briefly, after gently mixing the tubes by inversion, blood was centrifuged at 1,300*g* for 10 min at 20 °C. Supernatants were collected and immediately stored at −80 °C until analysis.

### TTR stabilization after oral administrations in humans

For TTR stabilization analysis, frozen plasma samples were thawed on ice. Samples were then mixed with a 8 M urea solution (in 40 mM sodium phosphate pH 7.4 and 80 mM KCl) to a final urea concentration of 4.8 M. One aliquot was taken immediately after adding urea (time 0) and the rest were incubated for 48 h at controlled RT conditions. After the incubation time points (0 or 48 h), TTR was crosslinked with 40 mM glutaraldehyde (2.5 M stock solution). The samples were quickly vortexed and incubated for 4 min at RT. Then, NaBH_4_ was added to a final concentration of 48 mM to quench the reaction. Samples were transferred to a measuring tube and read by immunoturbidity after 5 min at RT. Immunoturbidity was measured in a Beckman Coulter AU680 ISE instrument (Beckman Coulter), using the Olympus OSR6175 reagent and calibrator ODR3029 (Olympus) according to the manufacturer's instructions. Analysis was performed in triplicates. Informed consent was obtained from all subjects and the protocol was approved by the Ethics Committee of Hospital Vall d'Hebron (Barcelona).

### Tolcapone quantification in human plasma

Frozen plasma samples were thawed at RT and tolcapone levels were quantified by UPLC-MS/MS based on the methods described in refs 63, 64. Briefly, tolcapone and its internal standard (tolcapone-D_4_) were extracted from human plasma by methanol protein precipitation. Extracted samples were analysed on a Waters Acquity UPLC coupled with a Waters Xevo TQ MS triple quadrupole mass spectrometer. Chromatographic separation was achieved on an Acquity HSS C18 column using a gradient elution with the mobile phase of methanol and 0.1% formic acid in water. Analytes were monitored using electrospray-negative ionization mass spectrometry in the MRM mode (*m*/*z*=271.84>181 for tolcapone and 275.86>184.86 for tolcapone-D_4_).

## Additional information

**Accession codes:** The structures of WT-TTR and V122I-TTR complexed to tolcapone are deposited in the Protein Data Bank under accession codes PDB: 4D7B and PDB: 5A6I, respectively.

**How to cite this article:** Sant'Anna, R. *et al*. Repositioning tolcapone as a potent inhibitor of transthyretin amyloidogenesis and associated cellular toxicity. *Nat. Commun.* 7:10787 doi: 10.1038/ncomms10787 (2016).

## Supplementary Material

Supplementary Information Supplementary Figures 1-8 and Supplementary Tables 1-2.

## Figures and Tables

**Table 1:** Concentration of tolcapone in human plasma samples from treated individuals.

Subject	Tolcapone dose	Basal sample	Dosed sample*
Subject HV1	200 mg	<0.2 μg ml^−1^	3.7 μg ml^−1^ (13.6 μM)
Subject HV2	2 × 200 mg	<0.2 μg ml^−1^	20.6 μg ml^−1^ (75.6 μM)

^*^Dosed sample corresponds to plasma collected 2 h after drug administration (Tmax).
