# Author Correction: Repositioning tolcapone as a potent inhibitor of transthyretin amyloidogenesis and associated cellular toxicity

**DOI:** 10.1038/s41467-023-36239-z

**Published:** 2023-02-03

**Authors:** Ricardo Sant’Anna, Pablo Gallego, Lei Z. Robinson, Alda Pereira-Henriques, Nelson Ferreira, Francisca Pinheiro, Sebastian Esperante, Irantzu Pallares, Oscar Huertas, Maria Rosário Almeida, Natàlia Reixach, Raul Insa, Adrian Velazquez-Campoy, David Reverter, Núria Reig, Salvador Ventura

**Affiliations:** 1 Institut de Biotecnologia i Biomedicina and Departament de Bioquímica i Biologia Molecular, Universitat Autònoma de Barcelona, Bellaterra, Barcelona, 08193 Spain; 2 Molecular and Experimental Medicine Department, The Scripps Research Institute, La Jolla, California 92037 USA; 3 Molecular Neurobiology, IBMC- Instituto de Biologia Molecular e Celular i3S - Instituto de Investigação e Inovação em Saúde and ICBAS- Instituto de Ciências Biomédicas de Abel Salazar, Universidade do Porto, Porto, Portugal; 4 SOM-Biotech, Baldiri Reixac 4, Barcelona, 08028 Spain; 5 Institute of Biocomputation and Physics of Complex Systems (BIFI), Joint Unit IQFR-CSIC-BIFI, Universidad de Zaragoza, Zaragoza, 50018 Spain; 6 Department of Biochemistry and Molecular and Cell Biology, University of Zaragoza, Zaragoza, 50009 Spain; 7 Instituto de Investigación Sanitaria de Aragón (IIS Aragón), Zaragoza, 50009 Spain; 8 Fundación ARAID, Government of Aragón, Zaragoza, 50003 Spain

Correction to: *Nature Communications* 10.1038/ncomms10787, published online 23 February 2016

The original version of this Article contained the following errors:

The original version of this Article contained an error in the legend of Fig. 5c, which incorrectly omitted the following: ‘Please note that the same control was published in a previous article^65^ that reported the results obtained for other inhibitors of L55P-TTR aggregation that were tested in the same dot blot assay.’ This error has been corrected in both the PDF and HTML versions of the Article.

The original version of this Article omitted a reference to previous work in ‘Ferreira, N. et al. Molecular Tweezers Targeting Transthyretin Amyloidosis. *Neurotherapeutics* **11**, 450–461 (2014)’. This has been added as reference 65 in the legend of Fig. 5c, as described above. This error has been corrected in both the PDF and HTML versions of the Article.

In addition, the original version of the Supplementary Information associated with this Article contained errors in Supplementary Fig. 8, which showed an incomplete dot blot and was accompanied by an incomplete legend, which incorrectly read ‘Selected images of the dot blot used in main figure 5c.’ The correct version of the legend states ‘Uncropped blot for results shown in Figure 5c. Several compounds (including tolcapone, tafamidis, EGCG and CLR01) were tested in the same dot blot assay as potential inhibitors of L55P-TTR aggregation, sharing the same controls (no inhibitor) on an acetate cellulose membrane. Figure 5c showed the results obtained for the control, tolcapone and tafamidis, while a previous article showed the results obtained for the control, CLR01 and EGCG: Ferreira, N. et al. Molecular Tweezers Targeting Transthyretin Amyloidosis. *Neurotherapeutics* **11**, 450–461 (2014).’ The HTML has been updated to include a corrected version of the Supplementary Information; the original incorrect version of Supplementary Fig. 8 can be found as Supplementary Information associated with this Correction.

## Supplementary information

Incorrect Supplementary Figure 8

