# Supplementary material for: Repositioning tolcapone as a potent inhibitor of transthyretin amyloidogenesis and associated cellular toxicity
Source: Nat Commun. 2016 Feb 23;7:10787. doi: 10.1038/ncomms10787 (PMC4766415; doi:10.1038/ncomms10787)
Supplement: Supplementary Information — Supplementary Figures 1-8 and Supplementary Tables 1-2. [file ncomms10787-s1.pdf]

a

b

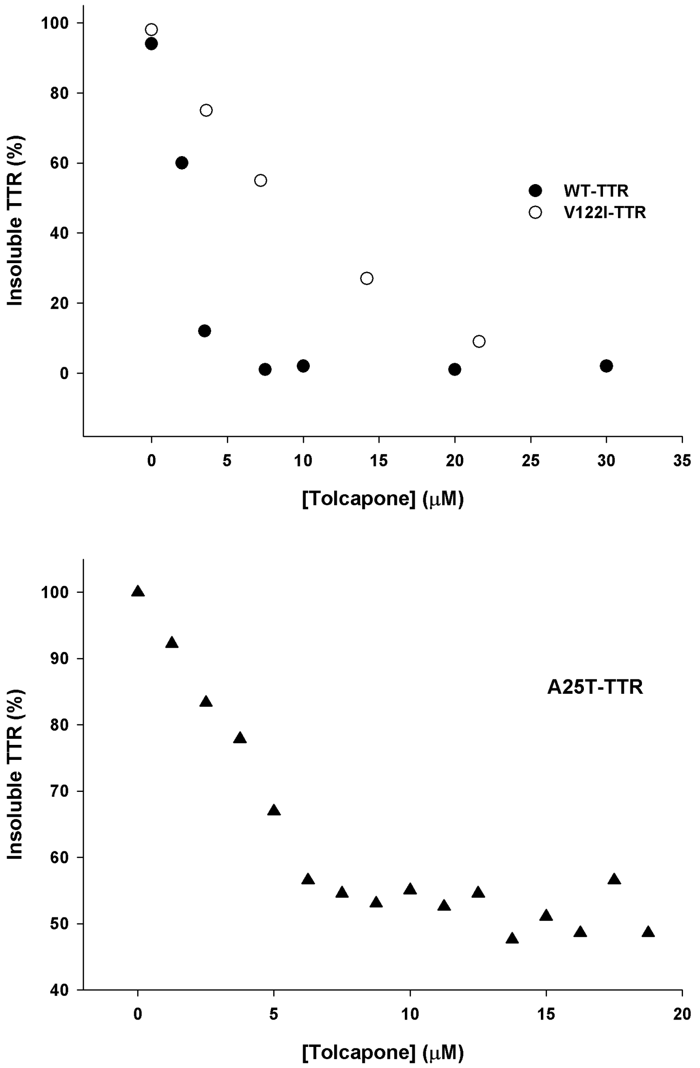

Supplementary Figure 1. Tolcapone prevents WT, V122I-TTR and A25T-TTR aggregation as measured by total protein precipitation.

a) WT and V122I-TTR were incubated with different concentrations of tolcapone under aggregation conditions (pH 4.4 and 37°C) for 72 hours. The samples were then centrifuged at 20,000 x g for 1 h at 4 °C and the supernatants (soluble protein) were carefully removed. The precipitated TTR from each sample was resolubilized by addition of 8 M guanidine solution followed by 1 h incubation at RT. TTR concentration was measured by spectroscopy at 280 nm and total amount of resolubilized TTR was calculated. The data is presented as % insoluble TTR with respect to samples in which no tolcapone were added (n = 1). b) The same experiment was performed for A25T-TTR at pH 5.0 and 37°C for 22 hours.

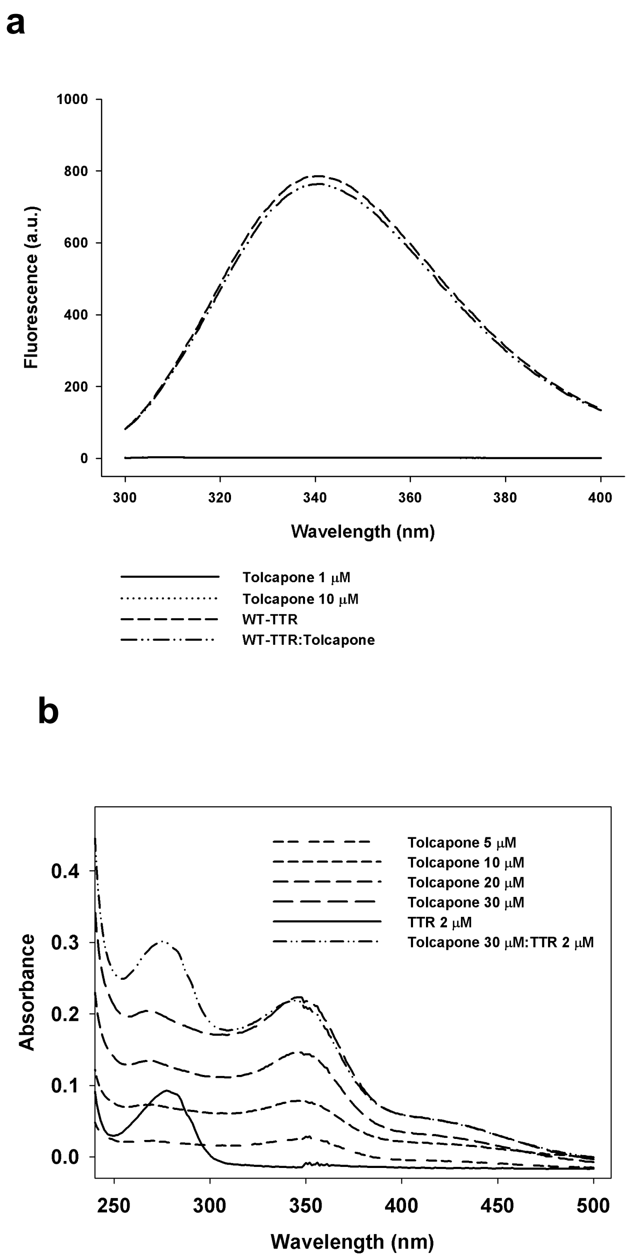

Supplementary Figure 2. Fluorescence and absorbance spectra of WT-TTR in the presence and absence of Tolcapone.

a) Fluorescence spectra of apo-WT-TTR, WT-TTR:tolcapone (2 μM:10 μM) and tolcapone alone (1 μM and 10 μM). Excitation wavelength 280 nm; emission collected from 300 to 400 nm. b) Absorbance spectra of tolcapone and of WT-TTR (2 μM) in the presence or absence of tolcapone.

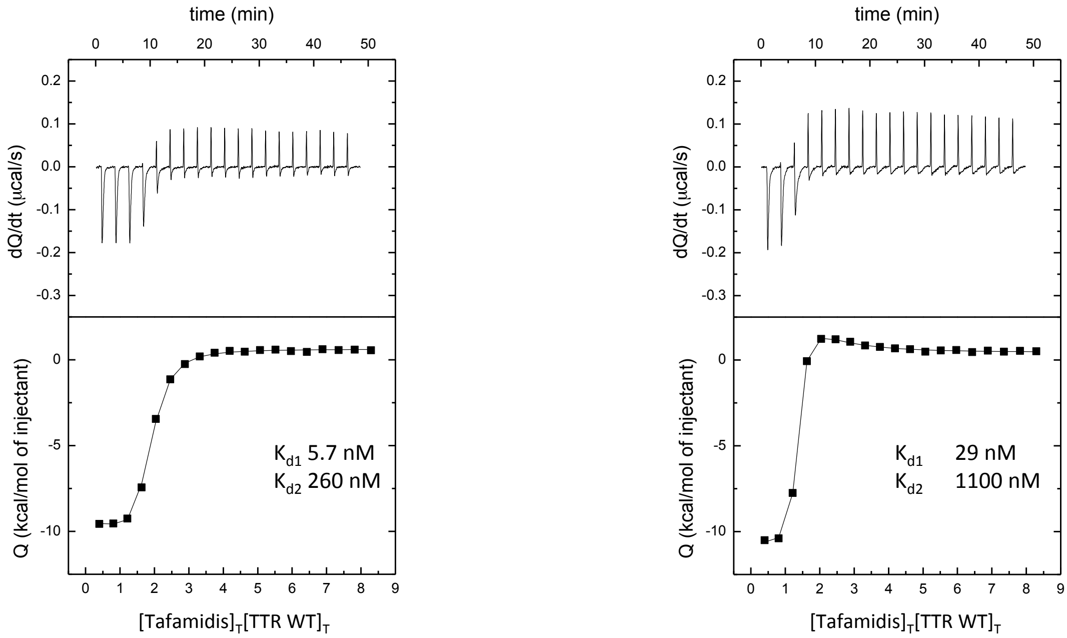

Supplementary Figure 3. Interaction of TTR with Tafamidis assessed by ITC.

WT (left) and V122I-TTR (right); upper panels: thermograms (thermal power versus time) after baseline correction; bottom panels: binding isotherm (normalized heat versus molar ratio of reactants).

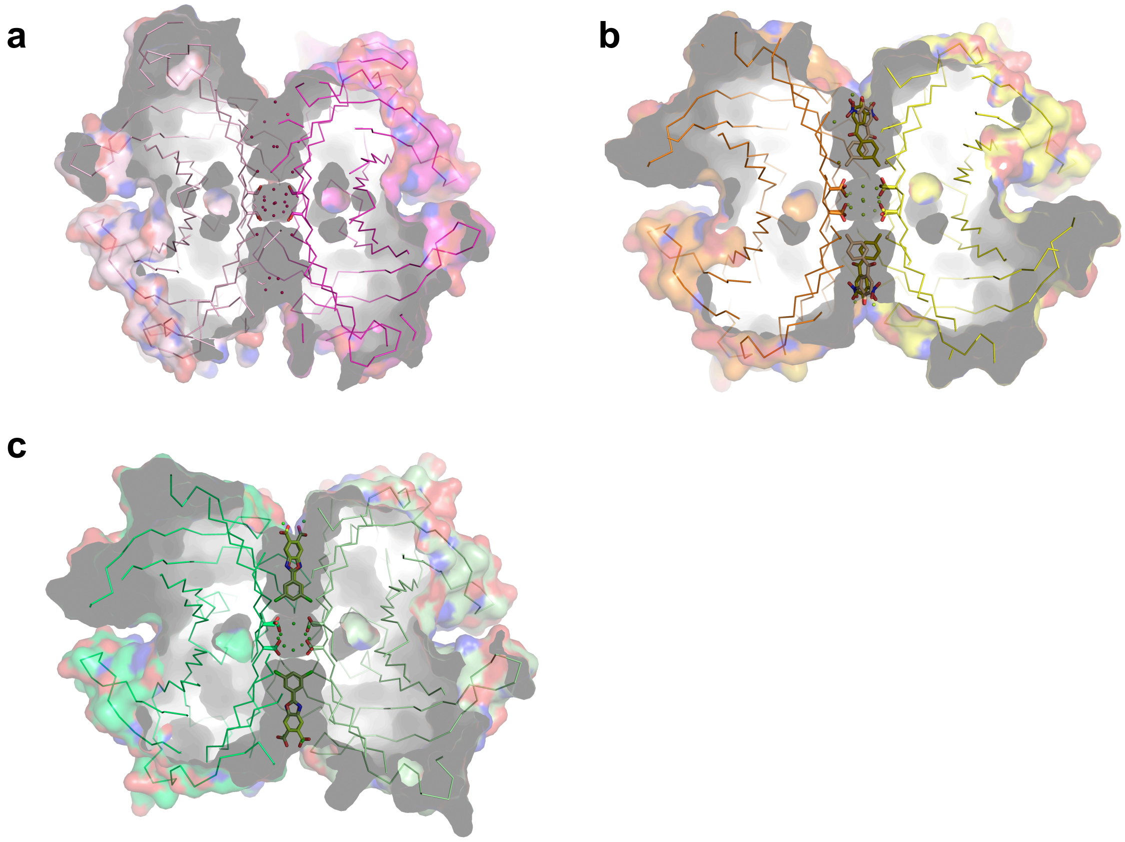

Supplementary Figure 4. TTR dimer interfaces.

a) Surface representation of the dimer in the WT TTR crystal structure (PDB: 1DVQ). b) Surface representation of the dimer interface in the tolcapone bound TTR crystal structure (PDB: 4D7B). c) Surface representation of the dimer interface in the tafamidis bound TTR crystal structure (PDB 3TCT). Dimer interface ordered water molecules in the crystal structures are represented by solid balls.

a

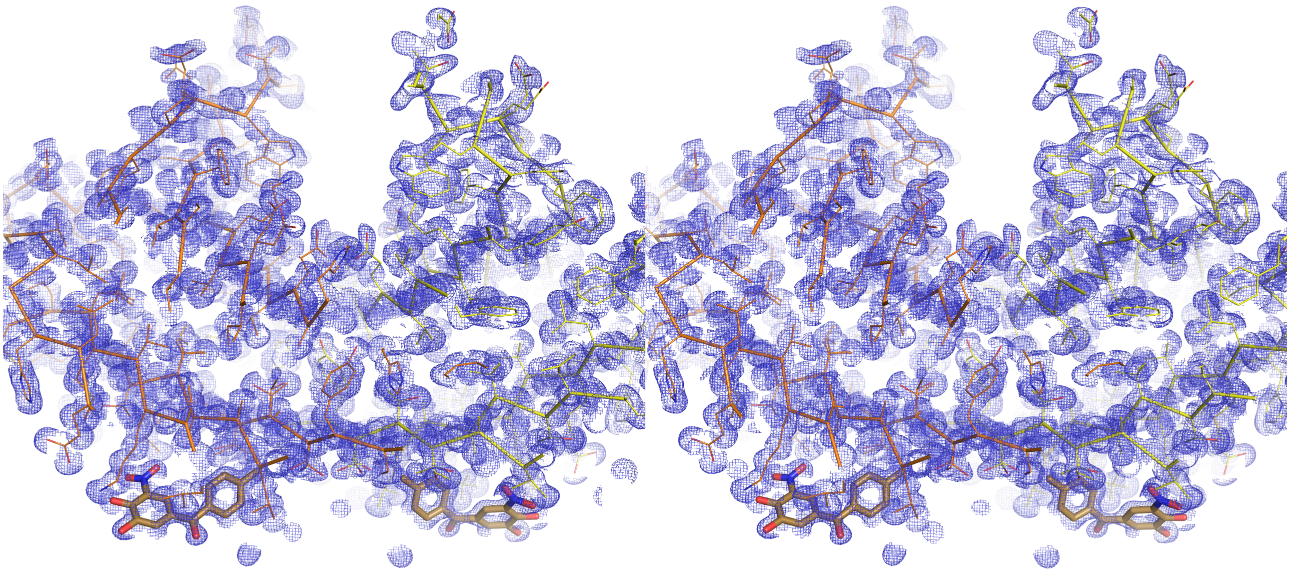

b

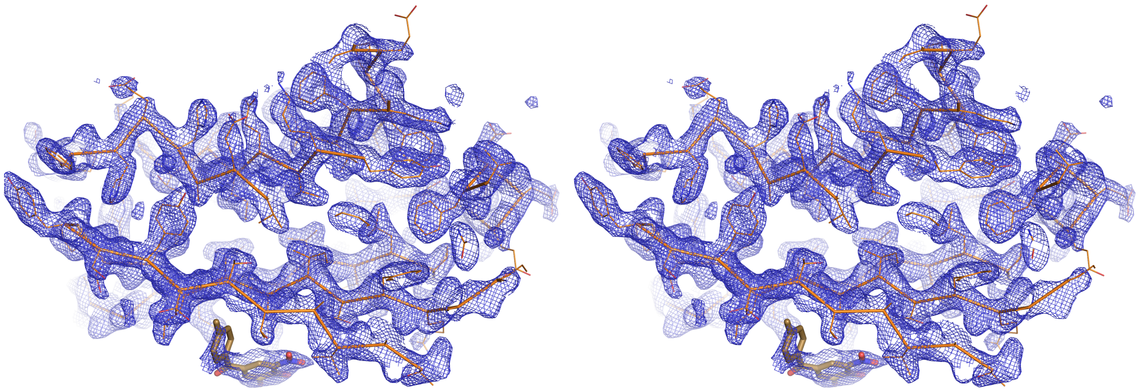

Supplementary Figure 5. Stereo images of the electron density maps.

Stereo views of the TTR wild type (a) and of the TTR-V122I (b) in complex with tolcapone with a 2Fo-Fc map (blue mesh) contoured at 1.5 σ.

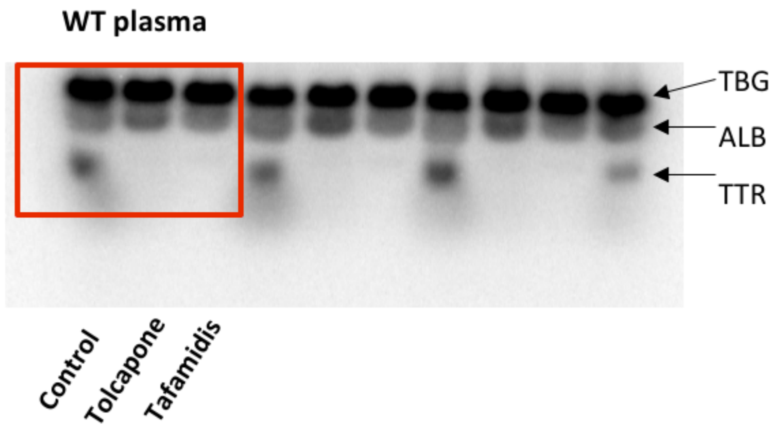

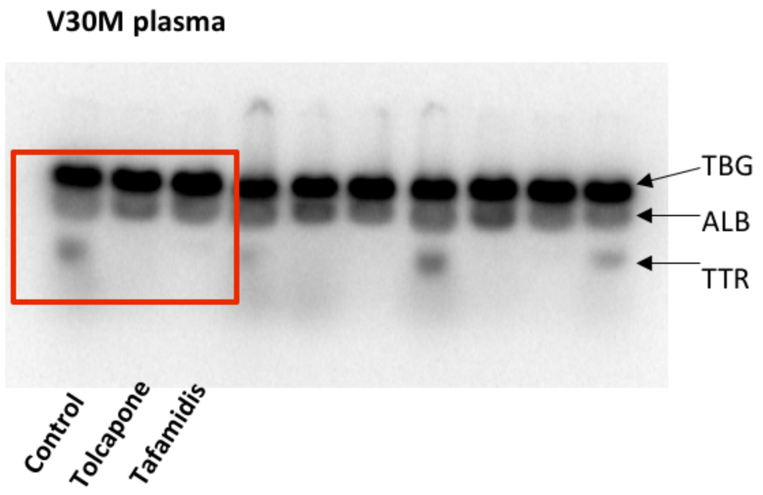

Supplementary Figure 6. Selected images of the film used in main figure 4a.

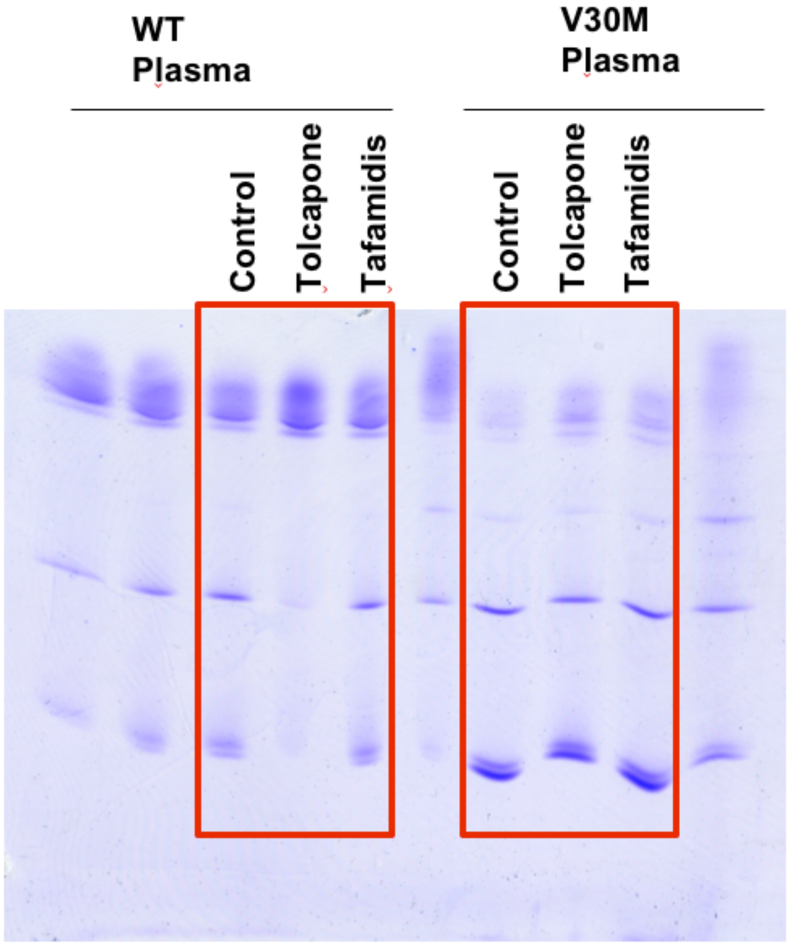

Supplementary Figure 7. Selected images of the gel used in main figure 4b.

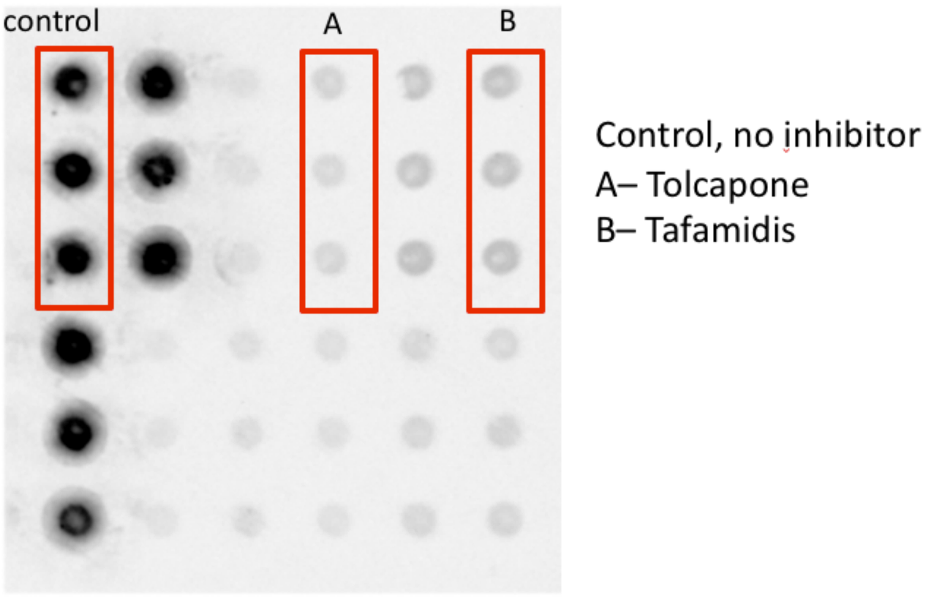

Supplementary Figure 8. Selected images of the dot blot used in main figure 5c.

Supplementary Table 1. Y78F-TTR aggregation inhibitory activity of selected compounds.

Compound	n	Model	EC50[a] (μM)	RA[b] (%)
**SOM01**	2	Linear	>100	-
**SOM02**	2	Linear	56	-
**SOM03**	2	Linear	>100	-
**SOM04**	2	Exponential	59	35.5
**SOM05**	2	Linear	>100	-
**SOM06**	2	Exponential	58	37.2
**SOM07**	4	Exponential	>100	26
**SOM08**	2	Linear	>100	-
**SOM09**	2	Linear	>100	-
**SOM10**	2	Linear	>100	-
**tolcapone**	4	Exponential	3.92	89.13
**SOM12**	2	Linear	>100	-
**SOM13**	2	Linear	70	-
**SOM14**	2	Linear	>100	-
**SOM15**	2	Linear	>100	-
**SOM16**	2	Exponential	>100	38.3
**SOM17**	2	Linear	>100	-
**SOM18**	2	Linear	84	-
**SOM19**	2	Linear	87.38	-
**SOM20**	2	Exponential	58	46.7
**SOM21**	2	Linear	78.61	-
**SOM22**	2	Linear	>100	-
**SOM23**	2	Linear	>100	-
**SOM24**	4	Exponential	>100	31.6
**SOM25**	2	Linear	>100	-
**SOM26**	2	Linear	>100	-
**SOM27**	2	Exponential	>100	34.9
**SOM28**	2	Exponential	>100	25.8
**SOM29**	2	Exponential	>100	24.6
**tafamidis**	6	Exponential	5.36	59.57

[a] EC_{50}: concentration of inhibitor at which the initial rate of TTR aggregation is one-half that of Y78F-TTR without inhibitor.

[b] RA (%): percent reduction of TTR aggregation rate at high inhibitor concentration relative to the rate in its absence.

Supplementary Table 2. Data collection and refinement statistics.

Data Collection	**TTR:TOLCAPONE**	**TTR V122I:TOLCAPONE**
Space group	$P2_12_12$	I222
Cell dimensions		
a, b, c (Å)	84.11, 43.81, 65.85	42.48, 62,55, 84,68
α, β, γ (°)	90, 90, 90	90, 90, 90
Resolution (Å)[a]	44 – 1.15 (1.21– 1.15)	50 – 1.86 (1.86 – 1.91)
R_{merge}[b]	0.051 (0.424)	0.040 (0.47)
I/σ_I	16.0 (3.6)	17.5 (2.4)
Completeness (%)	98.7 (96.6)	99.3 (100.0)
Redundancy	6.2 (5.3)	3.9 (3.9)
Refinement		
Resolution (Å)	44 - 1.15	50 – 1.86
No. Reflections	82563	38169
R_{work}/R_{free}[c]	17.09 / 20.06	19.70 / 27.08
No. Atoms	2153	942
Protein	1705	805
Ligand/ion	230	113
Water	221	24
B-factor		
Protein	20.40	49,40
Ligand/ion	15.40	56,90
Water	32.40	57,20
R.m.s deviations		
Bond lengths (Å)	0.031	0.016
Bond angles (°)	2.43	1.81
PDB code	4D7B	5A6I

[a]Statistic for highest resolution shell is shown in parentheses.

[b]$R_{merge} = \sum |I_i - \langle I \rangle| / \sum I_i$, where I_i is the ith measurement of the intensity of an individual reflection or its symmetry-equivalent reflections and <I> is the average intensity of that reflection and its symmetry-equivalent reflections.

[c]$R_{work} = \sum ||F_o| - |F_c|| / \sum |F_o|$ for all reflections and $R_{free} = \sum ||F_o| - |F_c|| / \sum |F_o|$, calculated based on the 5% of data excluded from refinement.
