# Supplementary figures and images for: Author Correction: Repositioning tolcapone as a potent inhibitor of transthyretin amyloidogenesis and associated cellular toxicity
Source: Nat Commun. 2023 Feb 3;14:582. doi: 10.1038/s41467-023-36239-z (PMC9898517; doi:10.1038/s41467-023-36239-z)

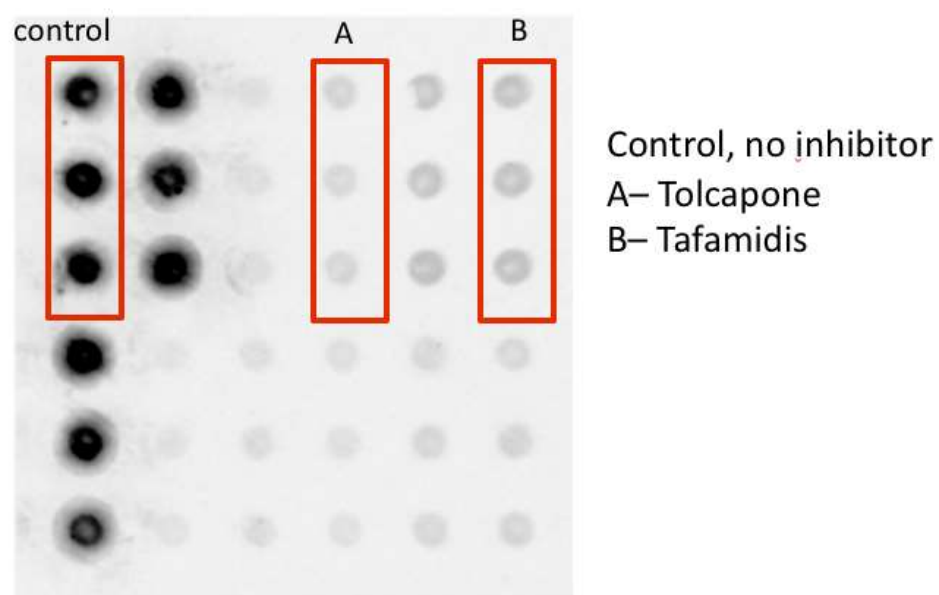

**Supplementary Figure 8. Selected images of the dot blot used in main figure 5c.**

Supplement: Supplementary file 1 — Incorrect Supplementary Figure 8 [file 41467_2023_36239_MOESM1_ESM.pdf]
